# DNA Binding Mode Analysis of a Core-Extended Naphthalene Diimide as a Conformation-Sensitive Fluorescent Probe of G-Quadruplex Structures

**DOI:** 10.3390/ijms221910624

**Published:** 2021-09-30

**Authors:** Chiara Platella, Rosa Gaglione, Ettore Napolitano, Angela Arciello, Valentina Pirota, Filippo Doria, Domenica Musumeci, Daniela Montesarchio

**Affiliations:** 1 Department of Chemical Sciences, University of Naples Federico II, 80126 Naples, Italy; chiara.platella@unina.it (C.P.); rosa.gaglione@unina.it (R.G.); ettore.napolitano@unina.it (E.N.); angela.arciello@unina.it (A.A.); domenica.musumeci@unina.it (D.M.); 2 Istituto Nazionale di Biostrutture e Biosistemi (INBB), 00136 Rome, Italy; 3 Department of Chemistry, University of Pavia, 27100 Pavia, Italy; valentina.pirota@unipv.it (V.P.); filippo.doria@unipv.it (F.D.); 4 Institute of Biostructures and Bioimaging (IBB) of the National Research Council (CNR), 80145 Naples, Italy

**Keywords:** G-quadruplex, naphthalene diimide, conformation-sensitive detection, fluorescent probe

## Abstract

G-quadruplex existence was proved in cells by using both antibodies and small molecule fluorescent probes. However, the G-quadruplex probes designed thus far are structure- but not conformation-specific. Recently, a core-extended naphthalene diimide (**c_ex_-NDI**) was designed and found to provide fluorescent signals of markedly different intensities when bound to G-quadruplexes of different conformations or duplexes. Aiming at evaluating how the fluorescence behaviour of this compound is associated with specific binding modes to the different DNA targets, **c_ex_-NDI** was here studied in its interaction with hybrid G-quadruplex, parallel G-quadruplex, and B-DNA duplex models by biophysical techniques, molecular docking, and biological assays. **c_ex_-NDI** showed different binding modes associated with different amounts of stacking interactions with the three DNA targets. The preferential binding sites were the groove, outer quartet, or intercalative site of the hybrid G-quadruplex, parallel G-quadruplex, and B-DNA duplex, respectively. Interestingly, our data show that the fluorescence intensity of DNA-bound **c_ex_-NDI** correlates with the amount of stacking interactions formed by the ligand with each DNA target, thus providing the rationale behind the conformation-sensitive properties of **c_ex_-NDI** and supporting its use as a fluorescent probe of G-quadruplex structures. Notably, biological assays proved that **c_ex_-NDI** mainly localizes in the G-quadruplex-rich nuclei of cancer cells.

## 1. Introduction

G-quadruplex structures are structural motifs of DNA or RNA, whose main unit involves a cyclic planar array of four guanines, which is called G-quartet [1,2,3,4]. One or more G-quartets can stack on each other, thus forming the three-dimensional architecture of a G-quadruplex, characterised by a central cavity in which metal cations are typically hosted, providing further stabilisation to the whole structure [1,2,3,4]. A peculiar feature of G-quadruplexes is their remarkable structural polymorphism, which depends on strands stoichiometry and orientation, type of linking loops, and guanine residues conformation. Particularly, according to the relative orientation of the strands forming the G-quadruplex structure, different topologies, i.e., parallel, antiparallel or hybrid conformations, can be distinguished [1,2].

Crucial biological roles are associated with G-quadruplexes which indeed are mainly localised in regulatory regions of the genome involved in DNA replication, transcription, and genomic maintenance [4,5,6,7]. Even more notable is their role as anticancer targets as a consequence of the non-random presence of G-rich sequences potentially able to fold in G-quadruplexes at both oncogene promoters and telomeres [4,7,8,9,10,11,12,13,14]. The effective formation of G-quadruplexes in the genome was proved a few years ago in human cells by using a specific antibody able to selectively recognize G-quadruplex structures [15,16,17,18,19]. However, the debate is still open on whether these findings are the result of artefacts due to chromatin fixation or to the addition of antibodies that can favour the formation of G-quadruplexes, thus artificially affecting their in vivo existence [20,21,22]. Moreover, studies exploiting antibodies were focused on fixed cells where the structural dynamics of nucleic acids are limited and thus processes involving G-quadruplexes along with the related downstream cellular pathways cannot be fully understood [20,21,22].

To address these issues, as an alternative to experiments with antibodies, a valuable approach for in-cell G-quadruplex detection is the use of small-molecule probes. Indeed, small molecules allow studying G-quadruplexes in live cells and in real-time overcoming the drawbacks associated with chromatin fixation [20,21,22]. Most small-molecule probes designed for G-quadruplexes thus far change their emission intensity, showing either fluorescence enhancement or quenching effects upon binding to DNA [23,24,25,26,27,28,29,30,31]. Recently, a G-quadruplex small molecule probe based on triangulenium ions has been reported and its interaction with G-quadruplexes has been visualised in live cells using fluorescence lifetime imaging microscopy. In detail, this probe shows a significantly longer fluorescence lifetime when bound to G-quadruplex DNA compared to duplex or single-stranded DNA [20,32,33].

Moreover, a new imaging platform based on single-molecule detection was recently applied to demonstrate that G-quadruplex formation is cell cycle-dependent and the presence of G-quadruplexes is directly related to the biological processes of live cells, such as DNA replication and transcription [21].

**Figure 1:**
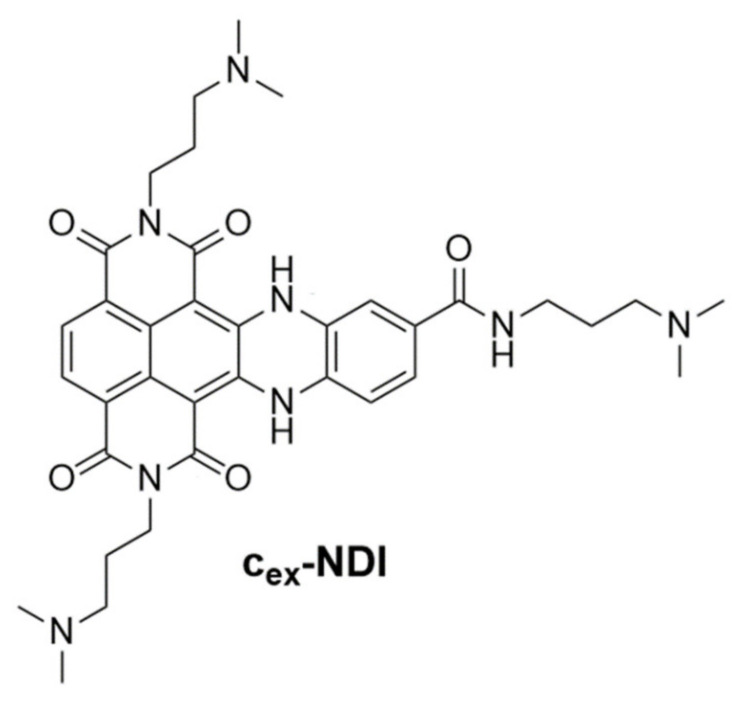
Chemical structure of the here investigated core-extended naphthalene diimide **c_ex_-NDI**.

However, both antibodies and small molecules designed thus far to target G-quadruplexes are structure-specific, i.e., able to selectively recognize G-quadruplexes over duplex structures, but not conformation-specific, i.e., able to discriminate G-quadruplexes vs. duplexes but also G-quadruplexes of different conformations. In this frame, recently, a core-extended naphthalene diimide (hereafter named **c_ex_-NDI**, Figure 1) was designed and proved able to provide fluorescent signals of different intensities when bound to G-quadruplexes of different conformations or duplex structures, thus acting both as a structure- and conformation-specific small-molecule probe for G-quadruplexes [34]. In detail, its interaction with hybrid/antiparallel G-quadruplexes, parallel G-quadruplexes, and duplex B-DNA was associated with high, medium, and low fluorescence quantum yields in solution, respectively [34]. Interestingly, a similar behaviour was found when this molecule was tested on Controlled Pore Glass supports, functionalised with G-quadruplexes of different conformations or duplex structures [35]. A molecule endowed with these features is an appealing candidate for the conformation-sensitive detection of G-quadruplexes in the genome, which would be an important breakthrough in this field to get insight into the real conformations adopted by these structures both in vitro and in vivo. This crucial information would help to fully understand the biological functions of G-quadruplexes and their interactions with macromolecules and small molecules. Thus far, the unique properties of **c_ex_-NDI** have been analysed only in preliminary studies [34]. With the aim of evaluating how the fluorescence behaviour of this compound is useful for in-cell G-quadruplex detection and how it is associated with specific binding mode to the different DNA targets, in-depth analyses were here performed on **c_ex_-NDI** binding to secondary structure-forming oligonucleotides. Indeed, deeper information on the peculiar DNA binding properties of **c_ex_-NDI** could allow for the rationalising of the behaviour of this unique molecule and provide information for the design of improved analogues thereof.

More in detail, the interactions of **c_ex_-NDI** with G-quadruplex and duplex models were here investigated by exploiting several biophysical techniques in a combined approach, particularly NMR, dynamic light scattering (DLS), gel electrophoresis, circular dichroism (CD), UV-vis, and fluorescence spectroscopies. Two G-quadruplex-forming oligonucleotides featured by hybrid or parallel topology, as well as a duplex-forming oligonucleotide characterised by a B-DNA conformation, were used as the model DNA targets. Moreover, docking studies were performed in order to build molecular models of the complexes formed between **c_ex_-NDI** and the same targets investigated experimentally. Finally, biological assays were carried out to assess the cytotoxicity of **c_ex_-NDI**, as well as evaluate its cellular entry ability and location in cells.

## 2. Results and Discussion

### 2.1. NMR Experiments

The interactions of **c_ex_-NDI** with hybrid and parallel G-quadruplexes, as well as a B-DNA duplex, were studied in a buffer mimicking the intranuclear environment, i.e., 100 mM KCl and 20 mM potassium phosphate buffer (pH 7.0). The three oligonucleotide sequences here investigated were selected for being structural models of the G-quadruplex or duplexes well-characterised by NMR, as well as well-known and validated models of DNA hybrid/parallel G-quadruplexes and duplexes [36,37,38]. In detail, m-tel24 and M2 oligonucleotides carrying the sequences d[TTGGG(TTAGGG)_3_A] and d(TAGGGACGGGCGGGCAGGGT) were chosen as models of hybrid and parallel G-quadruplexes, respectively [36,37]. As previously described [39], under the here used conditions, m-tel24 adopts a hybrid-1 G-quadruplex structural topology, involving G3:G21:G17:G9, G4:G10:G16:G22, and G5:G11:G15:G23 quartets, whereby G3, G9, G15, G16 and G21 are in *syn* conformation along with the *N*-glycosidic bond and the other residues are in *anti*-conformation. The three quartets are connected by one propeller and two lateral T-T-A loops, while the 5’- and 3’-ends include T1-T2 and A24 flanking residues, respectively. On the other hand, M2 adopts a parallel G-quadruplex comprising G3:G8:G12:G17, G4:G9:G13:G18, and G5:G10:G14:G19 quartets, which are linked by three propeller loops involving A6-C7, C11 and C15-A16 residues, while the 5’- and 3’-ends include T1-A2 and T20 flanking residues, respectively [37,39]. In addition, the Dickerson dodecamer ds12 of sequence d[(CGCGAATTCGCG)_2_], which forms a self-complementary bimolecular duplex [38], was chosen as a model for B-DNA duplexes.

**Figure 2:**
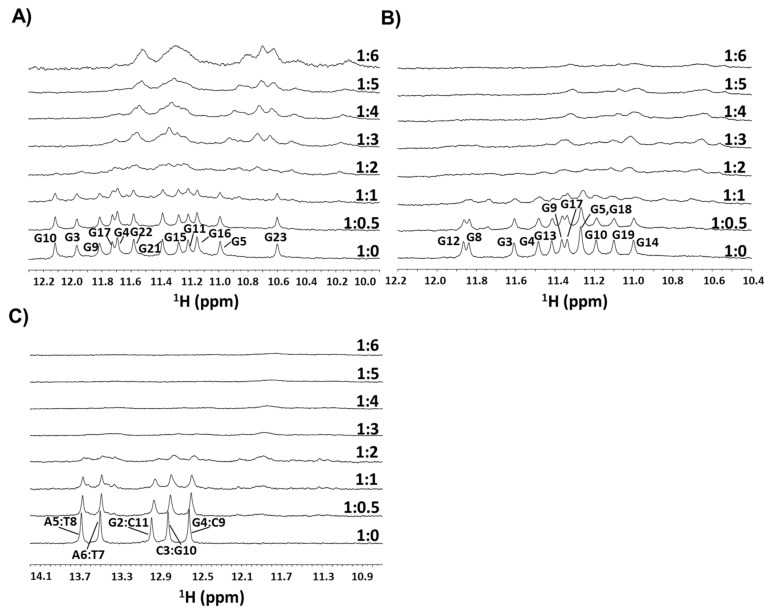
Imino proton regions of the ^1^H NMR spectra of (**A**) m-tel24 G-quadruplex, (**B**) M2 G-quadruplex and (**C**) ds12 duplex upon titration with **c_ex_-NDI** (from 0.5 to 6 equivalents). DNA/**c_ex_-NDI** ratios are shown on the right of the corresponding spectrum. Resonances for free G-quadruplexes and duplex were assigned according to refs. [39,41,42].

Under the specified conditions, all models gave high-quality NMR spectra, with the predominant G-quadruplex, for m-tel24 and M2, or duplex, in the case of ds12, with conformations accounting for >95% of the whole nucleotide material in the solution. More in detail, twelve narrow and well-resolved imino proton signals were observed in the range δ 10.5–12.2 ppm in the ^1^H NMR spectra of both m-tel24 and M2 G-quadruplexes (Figure 2A,B, bottom), consistent with the formation of G-quadruplexes with three stacked G-quartets [36,37,39,40]. On the other hand, five narrow and well-resolved imino proton signals were observed in the range δ 12.5–13.8 ppm of the ^1^H NMR spectrum of the ds12 duplex (Figure 2C, bottom), in accordance with previous reports for this self-complementary duplex consisting of twelve Watson-Crick base pairs [41,42,43].

Before studying the interactions of **c_ex_-NDI** with both G-quadruplex and duplex models, the ligand alone was analysed in solution by NMR (Figure S1). **c_ex_-NDI** gave ^1^H NMR signals in accordance with previous reports [34,39].

^1^H NMR titration experiments of m-tel24 G-quadruplex with **c_ex_-NDI** showed a stepwise broadening of the signals from 1:0 to 1:1 DNA/ligand ratio, accompanied with the appearance of novel, low-intensity signals at 1:1 DNA/ligand ratio at δ 10.85, 11.45, 11.64 and 11.75 ppm (Figure 2A). Then, a dramatic broadening of all imino signals was observed at 1:2 DNA/ligand ratio, while novel ^1^H NMR signals of m-tel24 appeared in the imino region upon increasing the **c_ex_-NDI** concentration (from 1:3 to 1:6 DNA/ligand ratio). Notably, these signals were up-field shifted compared to the free m-tel24 G-quadruplex and assigned to m-tel24 G-quadruplex/**c_ex_-NDI** complexes. Similar behaviour was observed in the aromatic and methyl regions of the ^1^H NMR titration spectra (Figures S2 and S3).

As far as the ^1^H NMR titration of M2 G-quadruplex with **c_ex_-NDI** is concerned, upon addition of 0.5 molar equivalents of **c_ex_-NDI**, slight broadening of the ^1^H NMR imino signals corresponding to the free M2 G-quadruplex was observed, together with the appearance of two novel signals at δ 11.74 and 11.77 ppm (Figure 2B). Further **c_ex_-NDI** additions from 1:1 to 1:2 DNA/ligand ratio caused a dramatic broadening of the whole set of imino signals. At 1:3 DNA/ligand, novel, broad imino signals appeared, up field shifted compared to the free M2 G-quadruplex and thus assigned to M2 G-quadruplex/**c_ex_-NDI** complexes. Further addition of **c_ex_-NDI** from 1:4 to 1:6 DNA/ligand ratio induced signal broadening, whereby at 1:6 DNA/ligand ratio all signals were hardly detectable from the baseline. On the other hand, a stepwise broadening was observed in the aromatic proton region of the M2 G-quadruplex titrated with **c_ex_-NDI** (Figure S4), while the gradual disappearance of methyl protons was accompanied by the appearance of a novel signal at δ 1.49 ppm observed at both 1:0.5 and 1:1 DNA/ligand ratio (Figure S5).

Finally, in the ^1^H NMR titration of ds12 duplex with **c_ex_-NDI**, stepwise broadening, as well as gradual upfield shift of proton signals compared to the free ds12 duplex was observed in imino (Figure 2C), aromatic (Figure S6) and methyl (Figure S7) regions from 1:0 to 1:2 DNA/ligand ratio, proving the interaction between **c_ex_-NDI** and ds12 duplex. Additionally, a novel signal was detected at δ 13.37 ppm at both 1:0.5 and 1:1 DNA/ligand ratio. On the other hand, ^1^H NMR signals were not detectable from 1:3 up to 1:6 DNA/ligand ratio.

Overall, the severe broadening of proton signals occurred at one molar ligand equivalent lower for M2 G-quadruplex (1:1 DNA/ligand ratio) than both m-tel24 G-quadruplex and ds12 duplex (1:2 DNA/ligand ratio). Moreover, even at high **c_ex_-NDI** concentration, no sharpening of ^1^H NMR signals was observed, indicating that complex equilibria, intermediate-to-fast on the NMR time scale, occurred for **c_ex_-NDI** with all the investigated G-quadruplex and duplex models [39,40].

Thus, the observed behaviour suggested that **c_ex_-NDI** could exhibit multiple binding poses on a single G-quadruplex/duplex unit and/or complexes of **c_ex_-NDI** with the examined targets comprising more than one DNA unit could be formed [39]. The low intensity of the ^1^H NMR signals observed for all the investigated systems in the imino as well as in the other spectral regions discouraged further, more in-depth NMR experiments.

### 2.2. DLS Experiments

**Figure 3:**
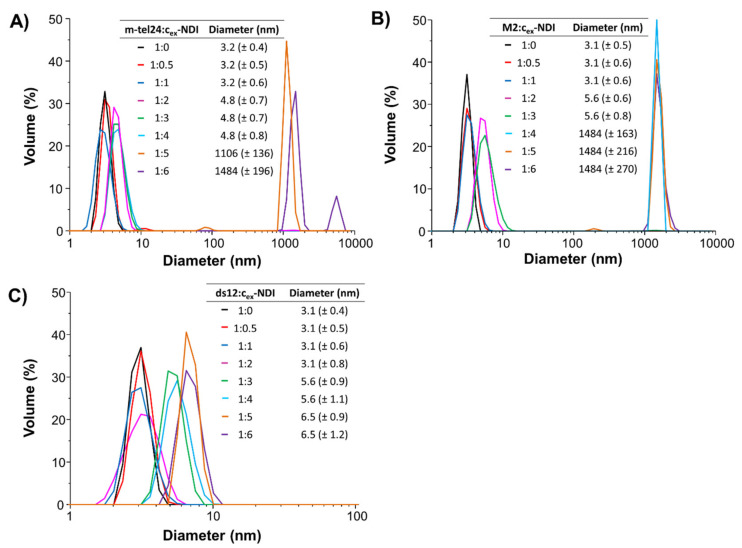
Volume-based particle size distribution for (**A**) m-tel24 G-quadruplex, (**B**) M2 G-quadruplex and (**C**) ds12 duplex in the absence and presence of different amounts of **c_ex_-NDI** (from 0.5 to 6 equivalents). Tables report the hydrodynamic size (±S.D.) for the different species formed on increasing **c_ex_-NDI** concentration.

In order to get information on the hydrodynamic size of DNA/**c_ex_-NDI** complexes, DLS experiments were performed. In detail, solutions of m-tel24 and M2 G-quadruplexes, as well as ds12 duplex, were titrated with increasing amounts of **c_ex_-NDI** (from 0.5 to 6 molar equivalents) and DLS analysis was carried out after equilibration of each sample (Figure 3). The volume-based particle size distribution showed the presence of a single species for both m-tel24 and M2 G-quadruplexes as well as ds12 duplex in the absence of the ligand, with hydrodynamic diameters of 3.2 (±0.4), 3.1 (±0.5), and 3.1 (±0.4) nm, respectively, in line with previous reports [39,44].

In the DLS titration of m-tel24 G-quadruplex with **c_ex_-NDI**, the addition of 0.5 or 1 molar equivalent of ligand did not affect m-tel24 G-quadruplex size (Figure 3A), indicating that the m-tel24/**c_ex_-NDI** complexes had the same hydrodynamic properties as m-tel24 G-quadruplex alone. On the other hand, the successive addition of 2, 3, and 4 molar ligand equivalents led to the formation of m-tel24 G-quadruplex/**c_ex_-NDI** complexes with a diameter of 4.8 (±0.7–0.8) nm, showing a drastic change in G-quadruplex size, which may be related to the formation of species comprising more than one ligand per single G-quadruplex unit and/or two G-quadruplex units whose interaction is mediated by **c_ex_-NDI** molecules [39]. From 5 to 6 molar **c_ex_-NDI** equivalents, species with even higher hydrodynamic diameter (1106–1484 nm) compatible with the formation of **c_ex_-NDI** aggregates were observed, including the molecules of non-bound ligand added in stoichiometric excess. Indeed, exploring the hydrodynamic behaviour of the ligand alone in the same range of concentrations as used in the previously described DLS titrations, **c_ex_-NDI** aggregates with hydrodynamic diameters higher than 1 µm were observed (Figure S8).

As far as M2 G-quadruplex is concerned, no significant variation of its hydrodynamic size was observed upon addition of 1 molar equivalent of ligand (Figure 3B), in analogy with the m-tel24 G-quadruplex/**c_ex_-NDI** system, indicating that M2 G-quadruplex preserved its monomeric fold, even when interacting with **c_ex_-NDI**, at low ligand concentration. On the other hand, higher size species formed at 1:2 and 1:3 M2 G-quadruplex/**c_ex_-NDI** ratios (5.6 ± 0.6–0.8 nm), suggesting that more than one ligand molecule could bind to the DNA target and/or a potential dimerisation of M2 G-quadruplex mediated by the ligand could occur. From 1:4 to 1:6 M2 G-quadruplex/**c_ex_-NDI** ratio, only very large species (ca. 1484 nm) were observed associated with the formation of **c_ex_-NDI** aggregates, as in the case of m-tel24 G-quadruplex/**c_ex_-NDI** system.

In the case of the ds12 duplex, no variation of DNA hydrodynamic diameter was observed up to 1:2 ds12 duplex/**c_ex_-NDI** ratio, while species with a size of 5.6 (±0.9–1.1) and 6.5 (±0.9–1.2) nm formed at 1:3/4 and 1:5/6 ds12 duplex/**c_ex_-NDI** ratio, respectively (Figure 3C). These findings suggested that up to 1:2 ds12 duplex/**c_ex_-NDI** ratio the overall size of the duplex model was not significantly affected by the bound ligand, while in the presence of additional two molar **c_ex_-NDI** equivalents a significant and gradual increase in complex size was observed. Moreover, no ligand aggregation was found in the DLS titration of the duplex model even when **c_ex_-NDI** was added at high ligand excess, probably because a higher number of ligand molecules should bind a single ds12 duplex than the other explored DNA models.

Notably, these results are fully consistent with the NMR titration experiments described above. Indeed, the ^1^H NMR signal broadening observed for all the DNA/**c_ex_-NDI** systems at ratios from 1:1 onwards can be well explained considering the formation of different DNA/**c_ex_-NDI** complexes, comprising one or more ligands and/or one or more DNA units, which are involved in intermediate-to-fast equilibria on the NMR time scale.

### 2.3. Gel Electrophoresis Experiments

**Figure 4:**
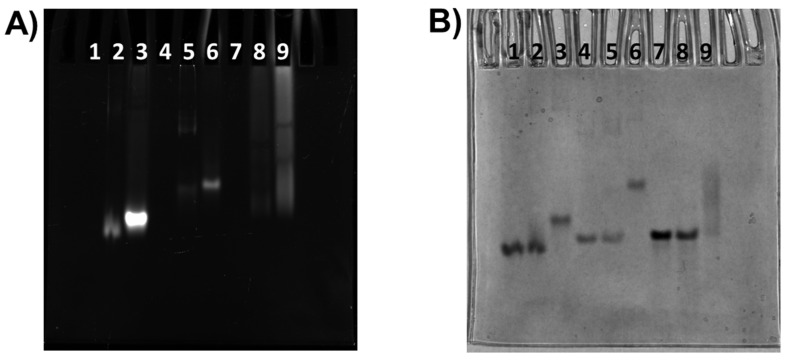
Native PAGE experiments. Oligonucleotide samples were loaded at 6 μM concentration in 100 mM aqueous KCl and 20 mM potassium phosphate buffer (pH 7) and resolved on 20% native PAGE. Lane 1: free m-tel24 G-quadruplex. Lane 2: m-tel24/**c_ex_-NDI**, 1:1. Lane 3: m-tel24/**c_ex_-NDI**, 1:6. Lane 4: free M2 G-quadruplex. Lane 5: M2/**c_ex_-NDI**, 1:1. Lane 6: M2/**c_ex_-NDI**, 1:6. Lane 7: free ds12 duplex. Lane 8: ds12/**c_ex_-NDI**, 1:1. Lane 9: ds12/**c_ex_-NDI**, 1:6. Gel was visualised by exploiting (**A**) the intrinsic fluorescence of the **c_ex_-NDI** and (**B**) Stains-All staining.

Native polyacrylamide gel electrophoresis (PAGE) experiments were carried out in order to obtain further information on the **c_ex_-NDI** binding to the G-quadruplex and duplex models. Oligonucleotides were analysed at 6 µM concentration in the absence or presence of different ligand amounts (1:1 and 1:6 DNA/**c_ex_-NDI** ratios) in 100 mM KCl and 20 mM potassium phosphate buffer (pH 7.0). The gel was visualised by both exploiting the intrinsic fluorescence of the **c_ex_-NDI** and the colorimetric staining by Stains-All, with the latter allowing only detection of the DNA bands (Figure 4). A single band was observed for all G-quadruplex and duplex models associated with the monomeric hybrid G-quadruplex, parallel G-quadruplex, and B-DNA conformations of m-tel24, M2, and ds12, respectively (Figure 4B).

Concerning the m-tel24 system (single band in lane 1, Figure 4B), at 1:1 DNA/ligand ratio, a slight retarded smear of the DNA band appeared compared to the DNA alone (lane 2, Figure 4B), in correspondence of which it was possible to observe the intrinsic fluorescence of the **c_ex_-NDI** (lane 2, Figure 4A). This behaviour can be explained considering the partial formation of the complex under these conditions with the equilibrium favouring the unbound species. The unbound DNA band completely disappeared (lane 3, Figure 4B) at a 1:6 DNA/ligand ratio with the equilibrium completely shifted towards the m-tel24 G-quadruplex/**c_ex_-NDI** complex; in this case, a single band was observed, with a perfect matching between the two used staining systems.

As far as the M2 system is concerned (single band in lane 4, Figure 4B), a slight reduction of the intensity of the unbound DNA was observed, accompanied by the appearance of small amounts of two retarded bands (lane 5, Figure 4B) containing **c_ex_-NDI** (lane 5, Figure 4A) and accounting for the formation of higher size species of M2 induced by the ligand. Also in this case, at 1:6 DNA/ligand ratio the equilibrium was completely shifted towards a single high size species, visualised as a single band by perfect overlapping of the two staining systems, whereas the unbound DNA band completely disappeared (lane 6, Figure 4A,B).

Finally, for the ds12 system (single band in lane 7, Figure 4B), no relevant variation of the mobility of the main band nor appearance of new retarded bands were detected at 1:1 DNA/ligand ratio (lane 8, Figure 4B) with the ligand fluorescence appearing as a diffuse band (lane 8, Figure 4A). In contrast, the free DNA band disappeared at 1:6 DNA/ligand ratio, and smeared retarded DNA bands (lane 9, Figure 4B) containing the fluorescent ligand (lane 9, Figure 4A) were observed. This behaviour proved that multiple higher size species of ds12 were present in equilibrium, bound to the ligand.

Overall, PAGE results were in line with DLS data, proving that species with higher size compared to the free G-quadruplex and duplex models were formed on increasing **c_ex_-NDI** molar equivalents.

### 2.4. CD and UV-Vis Experiments

CD and UV-Vis experiments were carried out by titrating solutions of each DNA model at a fixed concentration (i.e., 2 µM) in 100 mM KCl and 20 mM potassium phosphate buffer (pH 7.0) with increasing amounts of **c_ex_-NDI** (up to 6 molar equivalents).

**Figure 5:**
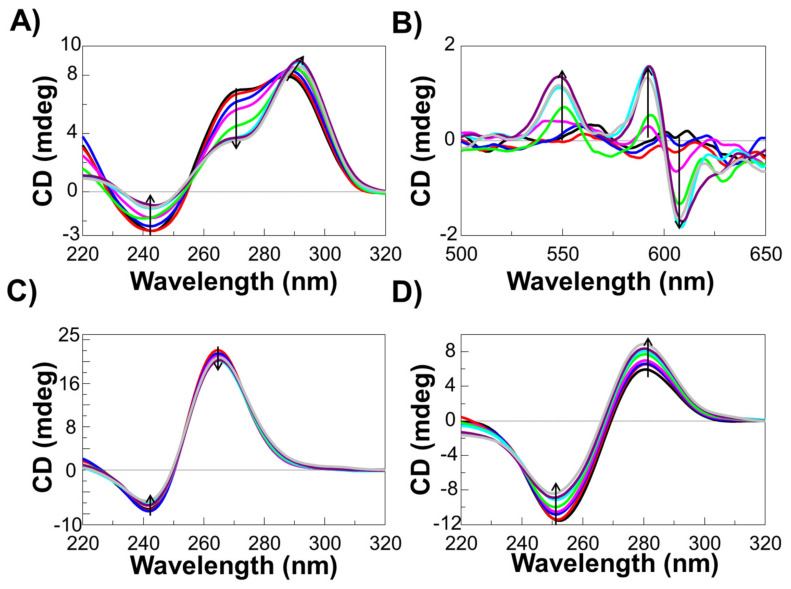
CD spectra of 2 μM solutions of (**A**,**B**) m-tel24 G-quadruplex, (**C**) M2 G-quadruplex and (**D**) ds12 duplex in 100 mM KCl and 20 mM potassium phosphate buffer (pH 7.0) in the absence or presence of increasing amounts (up to 6 molar equivalents) of **c_ex_-NDI**. The arrows indicate CD band intensity variation on increasing **c_ex_-NDI** concentration.

Regarding the CD studies, titration of m-tel24 G-quadruplex with **c_ex_-NDI** resulted in an increase of the CD band intensity centred at 287 nm accompanied with a red-shift of the band maximum up to 291 nm (Figure 5A). Additionally, reduction of CD intensity of the 270 nm shoulder as well as of the 243 nm minimum band was observed. According to PAGE analysis, these findings proved that the formation of higher size species of m-tel24 on increasing ligand concentration was also associated with relevant changes of the main conformation of m-tel24 G-quadruplex. Notably, induced CD bands were observed in correspondence of ligand absorption bands, centred at 550 and 600 nm (Figure 5B), which were related to binding of the ligand to the grooves of m-tel24 G-quadruplex, as also previously reported [34].

Titration of M2 G-quadruplex with **c_ex_-NDI** showed a decrease of the CD intensity absolute value for both the maximum band centred at 265 nm and the band minimum centred at 242 nm (Figure 5C), indicating that the parallel fold of the M2 G-quadruplex was preserved even upon ligand binding and consequent formation of higher size species.

Also in the case of the ds12 duplex, the overall secondary structure was not altered upon interaction with **c_ex_-NDI** and the formation of higher size species (Figure 5D). However, an increase and a decrease of the CD intensity absolute value were observed for the maximum band centred at 282 nm and the minimum band centred at 252 nm, respectively.

**Figure 6:**
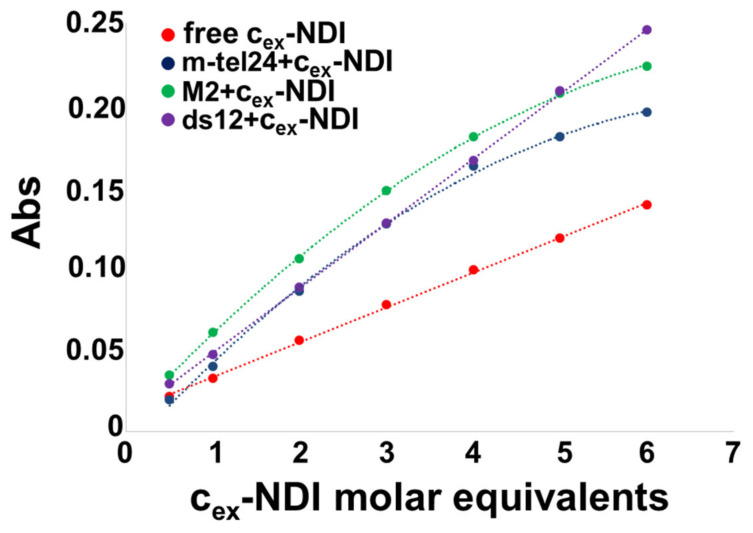
Absorbance at 597 nm as a function of the **c_ex_-NDI** molar equivalents for free **c_ex_-NDI** and for m-tel24 G-quadruplex, M2 G-quadruplex and ds12 duplex titrated with **c_ex_-NDI** (up to 6 molar equivalents).

As far as UV-vis titration experiments are concerned, comparing the absorbance values of the characteristic ligand band centred at 597 nm for the free ligand and ligand bound to each DNA model, it emerged that when the ligand was bound its absorbance was higher with all DNA models compared to the free ligand (Figure 6). In addition, a red-shift of the ligand band of 4 or 7 nm was associated with its binding to the G-quadruplexes and the duplex, respectively (Figure S9). Moreover, hyperbolic behaviour was observed with m-tel24 and M2 G-quadruplexes, while a linear increase of the absorbance vs. ligand concentration was found for the ds12 duplex (Figure 6), suggesting that a higher number of ligand molecules could bind a single ds12 duplex structure than m-tel24/M2 G-quadruplex to reach signal saturation.

### 2.5. Fluorescence Experiments

**Figure 7:**
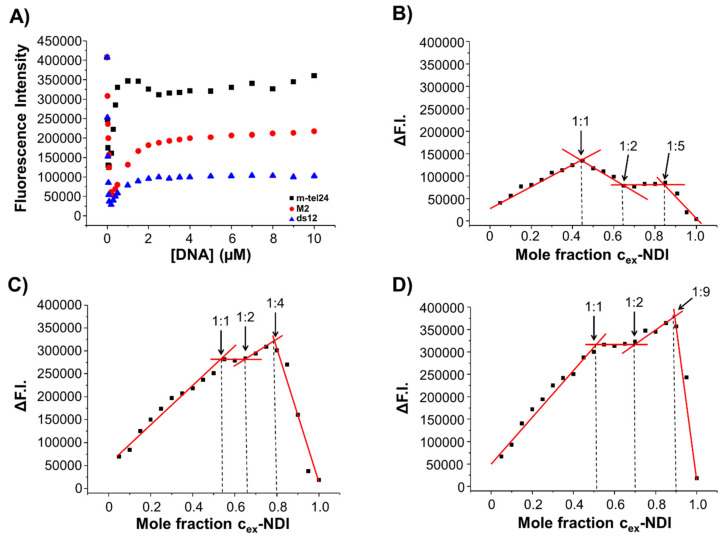
(**A**) Fluorescence titration experiments of **c_ex_-NDI** with m-tel24 G-quadruplex (black squares), M2 G-quadruplex (red circles) and ds12 duplex (blue triangles), and Job plot analysis for the binding of **c_ex_-NDI** to (**B**) m-tel24 G-quadruplex, (**C**) M2 G-quadruplex and (**D**) ds12 duplex. Labels in panels (**B**–**D**) indicate the stoichiometries of the different binding events observed for each system. ΔF.I. is the fluorescence intensity at each point from which the contribution of the ligand alone was subtracted.

To get further information about the binding mode and stoichiometry of the complexes formed between **c_ex_-NDI** and m-tel24 G-quadruplex, M2 G-quadruplex, and ds12 duplex, fluorescence experiments were performed. Fluorescence titration experiments were carried out by adding increasing amounts of each DNA to ligand solutions at 2 µM fixed concentration (Figure 7A and Figure S10A–C). A significant fluorescence quenching and a gradual red-shift of the intensity maxima were observed in all titration experiments from 0.02 to 0.2 µM DNA concentrations, corresponding to DNA/ligand concentration ratios from 0.01:1 to 0.1:1. On the other hand, a significant enhancement of fluorescence was observed from 0.2 to 10 µM DNA concentrations, i.e., moving from 0.1:1 DNA/ligand concentration ratio up to a DNA excess. Moreover, the fluorescence enhancement was also accompanied by a gradual redshift of the intensity maxima up to a 1:1 DNA/ligand ratio. These findings suggested that multiple binding events are involved in the interaction of **c_ex_-NDI** to the G-quadruplex and duplex models. In detail, it appears that when the ligand is in large excess, i.e., when large ligand aggregates are present and quenching effects are observed compared to the free ligand molecules [34], DNA addition results in a reduction of the fluorescence intensity probably due to the interaction of the DNA targets with the ligand aggregates, which quench even more the intrinsic fluorescence of the free ligand. On the contrary, when the DNA concentration becomes relevant in solution, ligand aggregates dissociate and simultaneously free ligand molecules can interact with the DNA target resulting in a different degree of fluorescence enhancement which is a function of the specific conformation and/or structure of the target. Indeed, at a 1:1 DNA/ligand ratio, i.e., when a plateau in fluorescence enhancement is reached for all the studied systems, the highest fluorescence intensity is observed when **c_ex_-NDI** interacts with the hybrid G-quadruplex model, a medium fluorescence intensity with the parallel G-quadruplex model and the lowest intensity with the duplex model, in line with previous reports [34,35].

In addition, fluorescence spectra were also recorded to obtain the Job plots for the different DNA/ligand mixtures, prepared by varying the **c_ex_-NDI** molar fraction from 0 to 1 and keeping constant the total molar concentration ([ligand] + [DNA]) (Figure 7B–D and Figure S11A–D). Job plot analysis for m-tel24 G-quadruplex/**c_ex_-NDI** mixtures showed changes at **c_ex_-NDI** molar fractions of 0.45, 0.64 and 0.84, corresponding to stoichiometries of approximately 1:1, 1:2 and 1:5 m-tel24 G-quadruplex/**c_ex_-NDI** (Figure 7B), while the analysis of the Job plot for M2 G-quadruplex/**c_ex_-NDI** mixtures showed changes at **c_ex_-NDI** molar fractions of 0.54, 0.65 and 0.80, corresponding to stoichiometries of approximately 1:1, 1:2 and 1:4 M2 G-quadruplex/**c_ex_-NDI** (Figure 7C). In the Job plot analysis for ds12 duplex, slope changes were observed at **c_ex_-NDI** molar fractions of 0.51, 0.70, and 0.90, corresponding to stoichiometries of approximately 1:1, 1:2, and 1:9 ds12 duplex/**c_ex_-NDI** (Figure 7D). These results evidenced that multiple binding events, and particularly three consecutive binding events, occurred in the interactions of **c_ex_-NDI** to both the G-quadruplex and duplex models, in agreement with NMR, DLS and fluorescence titrations data. Moreover, the presence and absence of aggregates observed by DLS measurements in the case of m-tel24/M2 G-quadruplexes and ds12 duplex systems, respectively, can be well explained considering the higher stoichiometry of the last binding event found for the ds12 duplex compared to the m-tel24 and M2 G-quadruplexes, which proves the ability of each duplex molecule to bind a higher number of **c_ex_-NDI** molecules than the studied G-quadruplex models. Analogously, the formation of aggregates at lower ligand concentration for M2 G-quadruplex than m-tel24 G-quadruplex is explained by the ability of m-tel24 G-quadruplex to form higher stoichiometry complexes in the last binding event with **c_ex_-NDI** than M2 G-quadruplex. Finally, for DNA/**c_ex_-NDI** ratios higher than 1:5, 1:4 and 1:9 for m-tel24 G-quadruplex, M2 G-quadruplex and ds12 duplex systems, respectively, significant fluorescence quenching (Figure 7B–D) accompanied by a blue-shift of the intensity maxima were observed (Figure S11B–D), further proving the formation of **c_ex_-NDI** aggregates when the ligand is in stoichiometric excess with respect to the highest number of **c_ex_-NDI** molecules that each target can potentially accommodate.

### 2.6. Docking Studies

To get a deeper insight into the binding mode of **c_ex_-NDI** to m-tel24 and M2 G-quadruplexes, as well as ds12 duplex, molecular docking studies were carried out in order to build structural models for the 1:1 DNA/ligand complexes.

**Figure 8:**
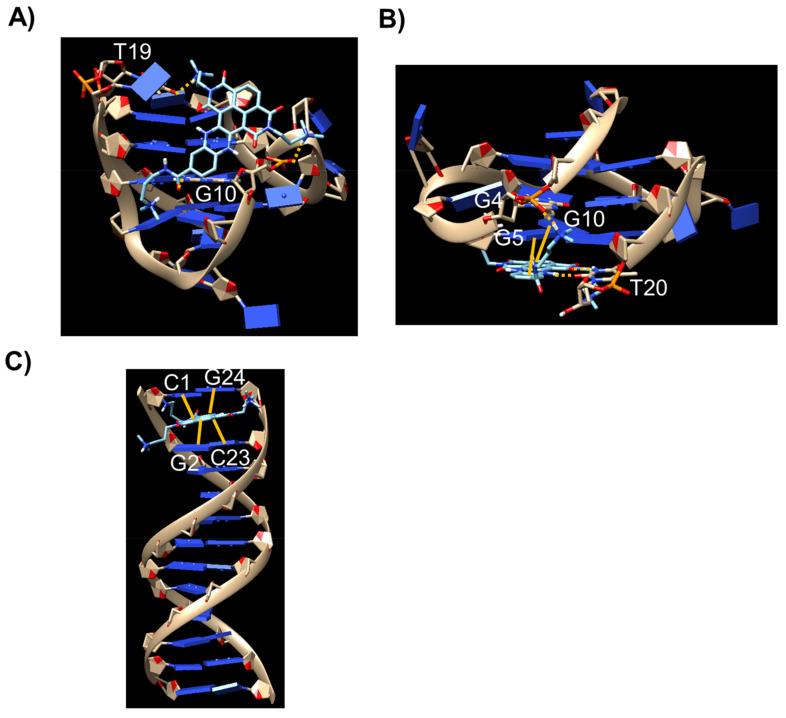
Binding mode of **c_ex_-NDI** when docked into (**A**) m-tel24 G-quadruplex, (**B**) M2 G-quadruplex and (**C**) ds12 duplex. **c_ex_-NDI** is represented as cyan sticks. Hydrogen bonds and electrostatic interactions are shown as yellow dashed lines while stacking interactions are shown as yellow bold lines. DNA residues involved in the interactions are indicated in each panel.

As far as the interaction of **c_ex_-NDI** with m-tel24 G-quadruplex is concerned, the most populated cluster involved the binding of the ligand to the groove between the second and third strands, forming the G-quadruplex (Figure 8A). In detail, the ligand was close to the 5’-end of the G-quadruplex where the groove has the highest accessibility. A binding energy of –7.5 kcal/mol was calculated for this binding pose of **c_ex_-NDI** onto m-tel24 G-quadruplex. Two hydrogen bonds between the protonated N13 of **c_ex_-NDI** and O4 of T19 as well as between amide oxygen of **c_ex_-NDI** and exocyclic NH_2_ of G10, together with an electrostatic interaction between protonated N9 of **c_ex_-NDI** and phosphate oxygen of G10 were observed.

On the other hand, for M2 G-quadruplex/**c_ex_-NDI** system, the most populated cluster involved the binding of the ligand to the 3’-end quartet (Figure 8B). The binding energy for this binding pose of **c_ex_-NDI** onto M2 G-quadruplex was −8.2 kcal/mol. π-π Stacking interactions of G5 and G10 with the aromatic rings of **c_ex_-NDI** were detected accompanied by two hydrogen bonds between one imide oxygen of **c_ex_-NDI** and H3 of T20 as well as between piperazine NH of **c_ex_-NDI** and O2 of T20, together with an electrostatic interaction between protonated N17 of **c_ex_-NDI** and a phosphate oxygen of G4.

As far as the interaction of **c_ex_-NDI** with ds12 duplex is concerned, the most populated cluster showed a pose involving the binding of the ligand via intercalation (Figure 8C), to which binding energy of −8.2 kcal/mol was associated. π-π Stacking interactions of both the C1:G24 and G2:C23 base pairs with the aromatic extended core of the ligand were observed.

Overall, docking results showed markedly different binding modes of **c_ex_-NDI** towards the G-quadruplex of different topologies and duplex structures. Indeed, binding to the groove was observed for the G-quadruplex with hybrid topology, binding to the outer quartet with the G-quadruplex parallel topology and intercalative binding mode with duplex DNA. The different binding modes and interactions can provide an explanation to the different fluorescence behaviour observed for **c_ex_-NDI** when bound to hybrid G-quadruplexes, parallel G-quadruplexes or duplex structures. In detail, it appears that the fluorescence intensity of DNA-bound **c_ex_-NDI** is a function of the amount of stacking interactions formed by the ligand with each DNA target. In particular, binding of **c_ex_-NDI** with hybrid G-quadruplex topology involves no stacking interactions and is featured by the highest fluorescence quantum yield, stacking of **c_ex_-NDI** on the parallel G-quadruplex topology is associated with intermediate quantum yield, and intercalative stacking of **c_ex_-NDI** as observed with duplex structures results in the lowest fluorescence quantum yield.

### 2.7. Biological Assays

#### 2.7.1. Analysis of the Effects of **c_ex_-NDI** on Cancer and Normal Breast Cell Lines

**Figure 9:**
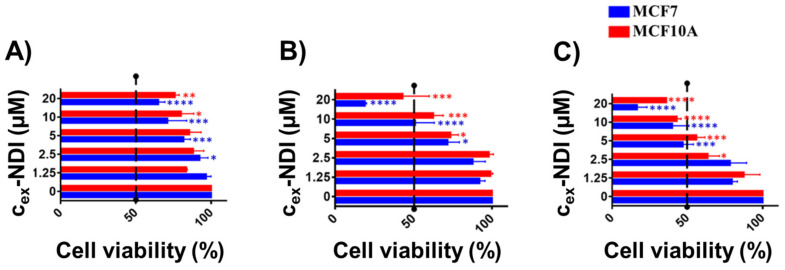
Effects of increasing concentrations of **c_ex_-NDI** (1.25–20 µM) on human breast MCF7 cells and non-tumorigenic human breast MCF10A cells upon (**A**) 24, (**B**) 48 and (**C**) 72 h incubation. Cell viability values are expressed as the percentage of cell viability obtained for treated vs. control cells grown in the absence of the molecule. Three independent experiments were performed, and, for all the experimental points, * *p* < 0.05, ** *p* < 0.01, *** *p* < 0.001 or **** *p* < 0.0001 were obtained for treated vs. control samples.

In order to select the optimal concentration and incubation time for the analysis of **c_ex_-NDI** internalisation in cancer and normal cells, the effects of **c_ex_-NDI** on the viability of both cancer human breast MCF7 cells and non-tumorigenic human breast MCF10A cells were preliminarily evaluated by the MTT assay. The obtained results indicated that **c_ex_-NDI** exerts significant dose- and time-dependent toxic effects on both cancer and normal cells (Figure 9A–C). IC_50_ values could be determined only after 48 and 72 h incubation, indicating that the two cell lines are similarly susceptible to the toxic effects of **c_ex_-NDI** (Table 1).

#### 2.7.2. Analysis of **c_ex_-NDI** Internalisation and Location into Cancer MCF7 and Normal MCF10A Cells by Confocal Laser Scanning Microscopy (CLSM)

**Figure 10:**
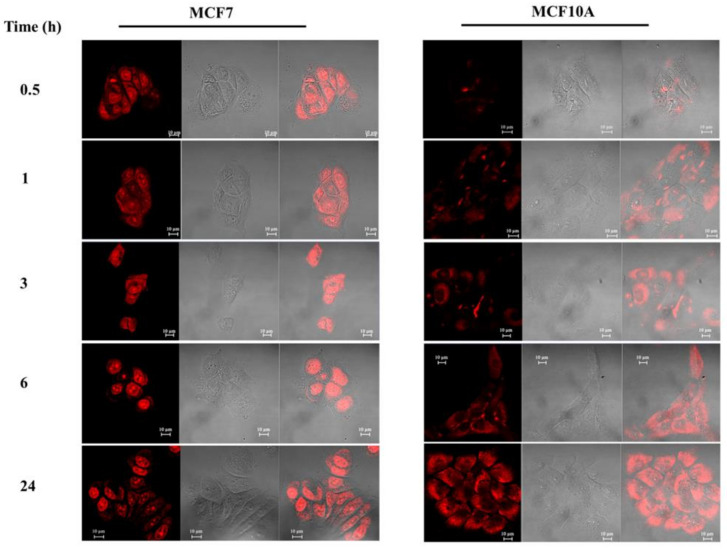
Internalisation of **c_ex_-NDI** into **MCF7** and **MCF10A** cells. Cells were cultured on glass coverslips in 24-well plates, grown to semi-confluency and incubated for 0.5, 1, 3, 6 and 24 h with 1.25 µM **c_ex_-NDI**. After fixing the cells with 4% paraformaldehyde, they were analysed by CLSM by using a 63x oil immersion objective. Scale bars correspond to 10 µm.

Based on the cell viability assay results, **c_ex_-NDI** internalisation was evaluated by CLSM analyses upon incubation of cancer MCF7 and normal MCF10A cells with a sub-toxic concentration of **c_ex_-NDI**, found to be 1.25 μM, and for 0.5, 1, 3, 6 and 24 h at 37 °C. As shown in Figure 10, the analyses, performed by using the settings suitable for Alexa Fluor 568 dye, revealed the internalisation of **c_ex_-NDI** into both cell lines even if with some differences. In detail, the intracellular fluorescence signal associated with **c_ex_-NDI** was found to progressively increase over time with a concomitant progressive accumulation in the nuclei upon 24 h incubation in the case of cancer MCF7 cells (Figure 10, left panels). Interestingly, **c_ex_-NDI** appeared to be mainly located in specific nuclear regions, which, in agreement with previous reports [31,45,46,47,48], can be associated with nucleoli. On the other hand, a lower fluorescence signal associated with **c_ex_-NDI** was observed in normal MCF10A cells. Particularly, significant **c_ex_-NDI** fluorescence was found only after 24 h incubation and, inside the normal cells, **c_ex_-NDI** appeared to be mainly located in the cytoplasm (Figure 10, right panels).

**Figure 11:**
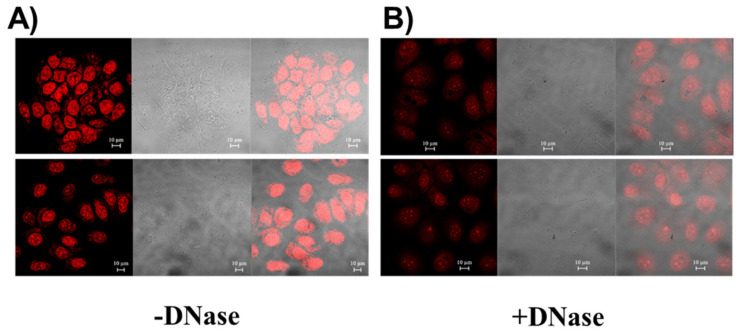
Binding of **c_ex_-NDI** to intracellular DNA of MCF7 cells. Cells were incubated with 1.25 µM **c_ex_-NDI** for 6 h and (**A**) fixed or (**B**) fixed and treated with 200 U of DNase.

In order to evaluate the ability of **c_ex_-NDI** to specifically target DNA in cancer cells, MCF7 cells were first incubated with the ligand and then treated with DNase (Figure 11). Notably, a significant reduction of fluorescence intensity was observed in the cells upon treatment with DNase, thus corroborating the capacity of **c_ex_-NDI** to selectively target the intracellular DNA of cancer cells (Figure 11 and Figure S12).

Considering the higher abundance of DNA G-quadruplexes in cancer than normal cell nuclei [16], these results proved that **c_ex_-NDI** can reach the nuclear G-quadruplex targets, thus resulting in high fluorescence intensity only when G-quadruplexes are significantly abundant in the examined cell line. On the contrary, only diffuse and less intense fluorescence signals associated with **c_ex_-NDI** can be observed in cells featured by a lower amount of DNA G-quadruplexes, with no signal inside the nuclei as a consequence of the quenching effect characteristic of **c_ex_-NDI** binding to duplex DNA.

## 3. Materials and Methods

### 3.1. Sample Preparation

DNA oligonucleotides were purchased from Biomers. The oligonucleotide concentration was determined by measuring the absorbance at 260 nm and 90 °C, using the appropriate molar extinction coefficients. **c_ex_-NDI** was synthesised and purified as previously described [34]. The **c_ex_-NDI** stock solution was prepared by dissolving the solid compound in aqueous solution at 100 mM KCl, 20 mM potassium phosphate buffer (pH 7), 90%/10% H_2_O/D_2_O, at 20 mM concentration of the ligand.

### 3.2. NMR Experiments

NMR data were collected on Bruker Avance 400 MHz NMR spectrometer at 25 °C. Oligonucleotide samples were prepared in 100 mM aqueous KCl, 20 mM potassium phosphate buffer (pH 7), 90%/10% H_2_O/D_2_O, at 0.2 mM oligonucleotide concentration per strand. In titration experiments, aliquots of the **c_ex_-NDI** stock solution were directly added to the oligonucleotide solutions inside the NMR tube. NMR spectra were acquired with the use of the DPFGSE solvent suppression method. DSS (4,4-dimethyl-4-silapentane-1-sulfonic acid) was used as a reference to calibrate the chemical shifts, assuming that DSS resonates at 0.0 ppm. NMR spectra were processed and analysed with the use of TopSpin 4.0.7 (Bruker).

### 3.3. DLS Experiments

DLS measurements were performed on a Zetasizer Nano ZS. Oligonucleotide samples were prepared at 0.2 mM concentration in 100 mM aqueous KCl and 20 mM potassium phosphate buffer (pH 7) and titrated with increasing amounts of **c_ex_-NDI**. The experiments were carried out at 25 °C at a scattering angle θ of 175° (backscatter detection). The diffusion coefficient of each population was calculated from the correlation function. The Stokes-Einstein equation was then used to evaluate the hydrodynamic diameter of free oligonucleotides, as well as of their complexes with **c_ex_-NDI**, from the related diffusion coefficients. Errors on hydrodynamic diameter were calculated as standard deviation of three independent measurements.

### 3.4. PAGE Experiments

The mixtures DNA/**c_ex_-NDI** (up to 1:6 ratio) and free DNA samples were loaded and analysed on native 20% polyacrylamide (19:1 acrylamide/bisacrylamide) gels. TBE 1X supplemented with 100 mM KCl was used as running buffer. The DNA samples loaded on gels were 6 µM in oligonucleotide concentration per strand in 100 mM aqueous KCl and 20 mM potassium phosphate buffer (pH 7). No migration marker was used. Samples were electrophoresed for 2 h at 100 V at room temperature. The bands were visualised by both exploiting the intrinsic fluorescence of the **c_ex_-NDI** and Stains-All staining.

### 3.5. CD and UV-Vis Experiments

CD spectra were recorded on a Jasco J-715 spectropolarimeter equipped with a Peltier-type temperature control system (model PTC-348WI), while UV-vis spectra were recorded on a JASCO V-550 UV-vis spectrophotometer equipped with a Peltier Thermostat JASCO ETC-505T. Quartz cuvettes with a path length of 1 cm were used. The spectra were recorded at 25 °C in the range 220–800 nm, with 200 nm/min scanning speed and 2.0 nm bandwidth, and they were corrected by subtraction of the background scan with buffer. CD spectra were averaged over three scans. The oligonucleotides were dissolved in 100 mM aqueous KCl and 20 mM potassium phosphate buffer (pH 7), to obtain 2 µM solutions. CD and UV-vis titrations were obtained by adding increasing amounts of the ligands (up to 6 molar equivalents, corresponding to a 12 µM solution in ligand) to m-tel24 G-quadruplex, M2 G-quadruplex and ds12 duplex solutions. After each ligand addition, the system was allowed equilibrating before recording the spectra.

### 3.6. Fluorescence Experiments

Fluorescence spectra were recorded at 20 °C on HORIBA JobinYvon Inc. FluoroMax®-4 spectrofluorometer equipped with F-3004 Sample Heater/Cooler Peltier Thermocouple Drive, by using a quartz cuvette with a 1 cm path length. The excitation wavelength of 555 nm was used and spectra were registered in the range 578–800 nm.

Fluorescence titration experiments were carried out at a fixed concentration (2.0 μM) of ligand. Increasing amounts of the m-tel24 and M2 G-quadruplexes, as well as the ds12 duplex (up to 10 μM concentration), were added from 120 μM stock solutions of each DNA sample dissolved in 100 mM aqueous KCl and 20 mM potassium phosphate buffer (pH 7). After each addition, the system was allowed equilibrating 10 min before recording the spectra.

For the construction of the Job plots, the molar fraction of **c_ex_-NDI** was varied from 0 to 1 and the total molar concentration ([ligand] + [DNA]) was kept constant at 2 μM.

### 3.7. Docking Studies

The NMR deposited structures of the G-quadruplex-forming oligonucleotides m-tel24 (PDB 2GKU) and M2 (PDB 5NYS) were used. The bimolecular duplex-forming oligonucleotide ds12 was prepared using as starting point the NMR deposited structure of ds12 (PDB 1NAJ) in which an intercalative binding site between C1:G24 and G2:C23 base pairs was created, using the crystallographic bimolecular duplex structure complexed with the intercalator ligand ellipticine as a template (PDB 1Z3F).

Molecular docking calculations were carried out using AutoDock Vina with the aid of its graphical user interface AutoDockTools [49]. The ligand **c_ex_-NDI** and DNA targets were prepared by use of AutoDockTools and UCSF Chimera by assigning bond orders, adding hydrogen atoms and generating the appropriate protonation states. The ligands and targets were then converted to proper Autodock PDBQT file formats and the Gaisteiger charges were assigned. The 3D grid box dimensions were defined including the whole DNA macromolecules. The docking area was centred on the DNA centre of mass and grid boxes of 80 Å × 80 Å × 70 Å, 84 Å × 62 Å × 86 Å and 62 Å × 120 Å × 60 Å for m-tel24 G-quadruplex, M2 G-quadruplex and ds12 duplex, respectively, with a 0.375 Å spacing, were used. One hundred docking poses were generated by using as docking parameters “seed = random”, “exhaustiveness = 24” and “number of binding modes = 20” for each of the five runs performed for each DNA/ligand system. Docking poses were clustered on the basis of their root-mean-square deviation and were ranked on the basis of binding energy. Molecular modelling figures were drawn by UCSF Chimera.

### 3.8. Biological Assays

#### 3.8.1. Cell Cultures and Cytotoxicity Assays

Human breast adenocarcinoma cells MCF7 were cultured in high-glucose Dulbecco’s modified Eagle’s medium (DMEM) supplemented with 10% foetal bovine serum and 1% penicillin-streptomycin at 37 °C in the presence of 5% CO_2_. Human non-tumorigenic epithelial cells from mammary gland ATCC-CRL-10317 MCF10A were cultured in complete Mammary Epithelial Growth Medium (MEGM) containing 100 ng/mL of cholera toxin at 37 °C in the presence of 5% CO_2_.

**c_ex_-NDI** effects on cells viability were evaluated by seeding cells into 96-well plates (100 µL/well) at a density of 3 × 10^3^ cells/well. Upon 24 h, cells were incubated with increasing molecule concentrations (0–20 µM), for 24, 48, or 72 h. At the end of the treatment, cell viability was assessed by the 3-(4,5-dimethylthiazol-2-yl)-2,5-diphenyltetrazolium bromide (MTT) assay. MTT reagent, dissolved in DMEM without phenol red, was added to the cells (100 µL/well) at a final concentration of 0.5 mg/mL. After 4 h at 37 °C, the culture medium was removed and the resulting formazan salts were dissolved by the addition of isopropanol containing 0.1 N HCl (100 µL/well) [50,51,52]. Absorbance values of blue formazan were determined at 570 nm by using an automatic plate reader (Synergy™ H4 Hybrid Microplate Reader, BioTek Instruments, Inc., Winooski, VT, USA). Cell survival was expressed as the percentage of viable cells in the presence of the molecule with respect to control cells.

#### 3.8.2. Internalisation of **c_ex_-NDI** into MCF7 and MCF10A Cells by CLSM Analyses

Analyses were performed as previously described [53,54]. To assess the healthy conditions of nuclei in the used MCF7 and MCF10A cells, a preliminary analysis of the cells was performed by incubating them with Hoechst (0.001 mg/mL) for 15 min at room temperature (RT) and visualising them by using a confocal laser-scanning microscope (CLSM) Zeiss LSM 700 and a 63x oil objective (Figure S13).

For the experiments with the ligand, MCF7 and MCF10A cells were seeded on glass coverslips in 24-well plates, grown to semi-confluency, and then incubated for 0.5, 1, 3, 6 and 24 h in the presence of 1.25 μM **c_ex_-NDI**. After the incubation, cells were washed with phosphate buffer saline (PBS) 1x and then fixed with 4% paraformaldehyde in PBS for 15 min at RT. Cells were then washed twice with PBS, mounted on coverslips and then observed by using a confocal laser-scanning microscope Zeiss LSM 700 and a 63x oil objective.

For the experiments with DNase, MCF7 cells were grown in DMEM supplemented with 10% heat-inactivated foetal bovine serum. Cells were maintained as a monolayer at 37 °C in a 5% CO_2_ humidified atmosphere. Cells were seeded onto glass coverslips in 24-well plates and incubated overnight; next, cells were treated with 1.25 μM **c_ex_-NDI** for 6 h at 37 °C, fixed with 4% paraformaldehyde in PBS for 20 min and then treated with 200 units DNase I. Cells were then washed twice with PBS, mounted on coverslips and then observed by using a confocal laser-scanning microscope Zeiss LSM 700 and a 63x oil objective.

#### 3.8.3. Statistical Analyses

Statistical analyses were performed by using Student’s *t* test. Significant differences were indicated as * (*p* < 0.05), ** (*p* < 0.01), *** (*p* < 0.001) or **** (*p* < 0.0001).

## 4. Conclusions

The interactions of the core-extended naphthalene diimide **c_ex_-NDI** with hybrid G-quadruplex, parallel G-quadruplex and B-DNA duplex models were investigated by using a combination of biophysical techniques and molecular modelling. **c_ex_-NDI** showed markedly different binding modes to the three examined DNA targets. When the ligand was in large excess, ligand aggregates formed and were able to bind the DNA molecules. On increasing DNA concentration, ligand aggregates dissociated and the free monomers bound the DNA targets showing three consecutive binding events. DNA/ligand complexes of 1:1 and 1:2 ratios were found for all the investigated targets; in turn, the last binding event was peculiar for each target and based on the ability of the latter to accommodate a different number of **c_ex_-NDI** molecules. Indeed, up to five, four and nine ligand molecules could bind to hybrid G-quadruplex, parallel G-quadruplex, and duplex models, respectively. Due to the typically low concentration of small molecules reaching the G-quadruplexes in the cell nuclei, molecular models were built considering only the 1:1 binding event which is the most plausible one in the cellular environment. The preferential binding sites of the ligand in 1:1 DNA/ligand complexes were the groove and the outer quartet for the hybrid G-quadruplex and the parallel G-quadruplex, respectively, whereas the preferred site for the duplex was the intercalative site. Hence, different interactions were found between the ligand and each DNA target, with stacking interactions absent or present in medium or high amounts for the hybrid G-quadruplex, parallel G-quadruplex, and duplex, respectively. The different binding modes and interactions explain the different fluorescence behaviour of **c_ex_-NDI** in the recognition of G-quadruplexes featured by different conformations and duplex structures, providing the rationale behind the conformation-sensitive properties of **c_ex_-NDI** as a fluorescent probe of G-quadruplex structures. A lower amount of stacking interactions results in higher fluorescence enhancement upon **c_ex_-NDI** binding to DNA. In detail, the interaction with hybrid G-quadruplex topology, involving no stacking interactions, is featured by the highest fluorescence quantum yield, while the stacking of **c_ex_-NDI** on the parallel G-quadruplex topology is associated with intermediate quantum yield. Finally, the intercalative stacking of **c_ex_-NDI** - as observed with duplex structures - results in the lowest fluorescence quantum yield experimentally found. Interestingly, biological assays proved that **c_ex_-NDI** easily enters both cancer and normal cells. However, the higher fluorescence intensity in the nuclei was observed for cancer than normal cells, which can be well explained considering the higher abundance of G-quadruplex structures in cancer than in normal cell nuclei, definitely validating the ability of **c_ex_-NDI** to reach the DNA G-quadruplex targets.

Altogether these findings provide precious information for the design of improved analogues of **c_ex_-NDI** in the context of developing more efficient conformation-sensitive fluorescent probes of G-quadruplex structures, to be studied and tested in cells by fluorescence lifetime imaging microscopy.

## Figures and Tables

**Table 1:** IC_50_ values were determined by testing increasing concentrations of **c_ex_-NDI** for different time intervals on MCF7 and MCF10A cells by MTT assays. N/A: not applicable.

IC_50_ (µM)					
MCF7			MCF10A		
24 h	48 h	72 h	24 h	48 h	72 h
N/A	12	9	N/A	16	11

## Data Availability

Not applicable.

## Supplementary Materials

The following are available online at https://www.mdpi.com/article/10.3390/ijms221910624/s1, Figures S1–S7: NMR experiments, Figure S8: DLS experiments, Figure S9: UV-vis experiments, Figures S10–S11: Fluorescence experiments, Figures S12–S13: Confocal microscopy experiments.
