# Supplementary material for: DNA Binding Mode Analysis of a Core-Extended Naphthalene Diimide as a Conformation-Sensitive Fluorescent Probe of G-Quadruplex Structures
Source: Int J Mol Sci. 2021 Sep 30;22(19):10624. doi: 10.3390/ijms221910624 (PMC8508963; doi:10.3390/ijms221910624)
Supplement: Supplementary file 1 [file ijms-22-10624-s001.zip › ijms-1340537-supplementary.pdf]

Supplementary Information

to the manuscript:

DNA binding mode analysis of a core-extended naphthalene diimide as a conformation-sensitive fluorescent probe of G-quadruplex structures

Chiara Platella [1], Rosa Gaglione [1], Ettore Napolitano [1], Angela Arciello [1], Valentina Pirota [2], Filippo Doria [2], Domenica Musumeci [1,3], Daniela Montesarchio [1,*]

[1]*Department of Chemical Sciences, University of Naples Federico II, via Cintia 21, 80126 Naples, Italy*
[3]*Department of Chemistry, University of Pavia, Viale Taramelli 12, 27100 Pavia, Italy*
[3]*Institute of Biostructures and Bioimaging (IBB) of the National Research Council (CNR), Napoli*

* Correspondence: daniela.montesarchio@unina.it

Present Address: Daniela Montesarchio, Department of Chemical Sciences, University of Naples Federico II, via Cintia 21, I-80126 Naples, Italy

Tel: +39 081 674126; Fax: +39 081 674313

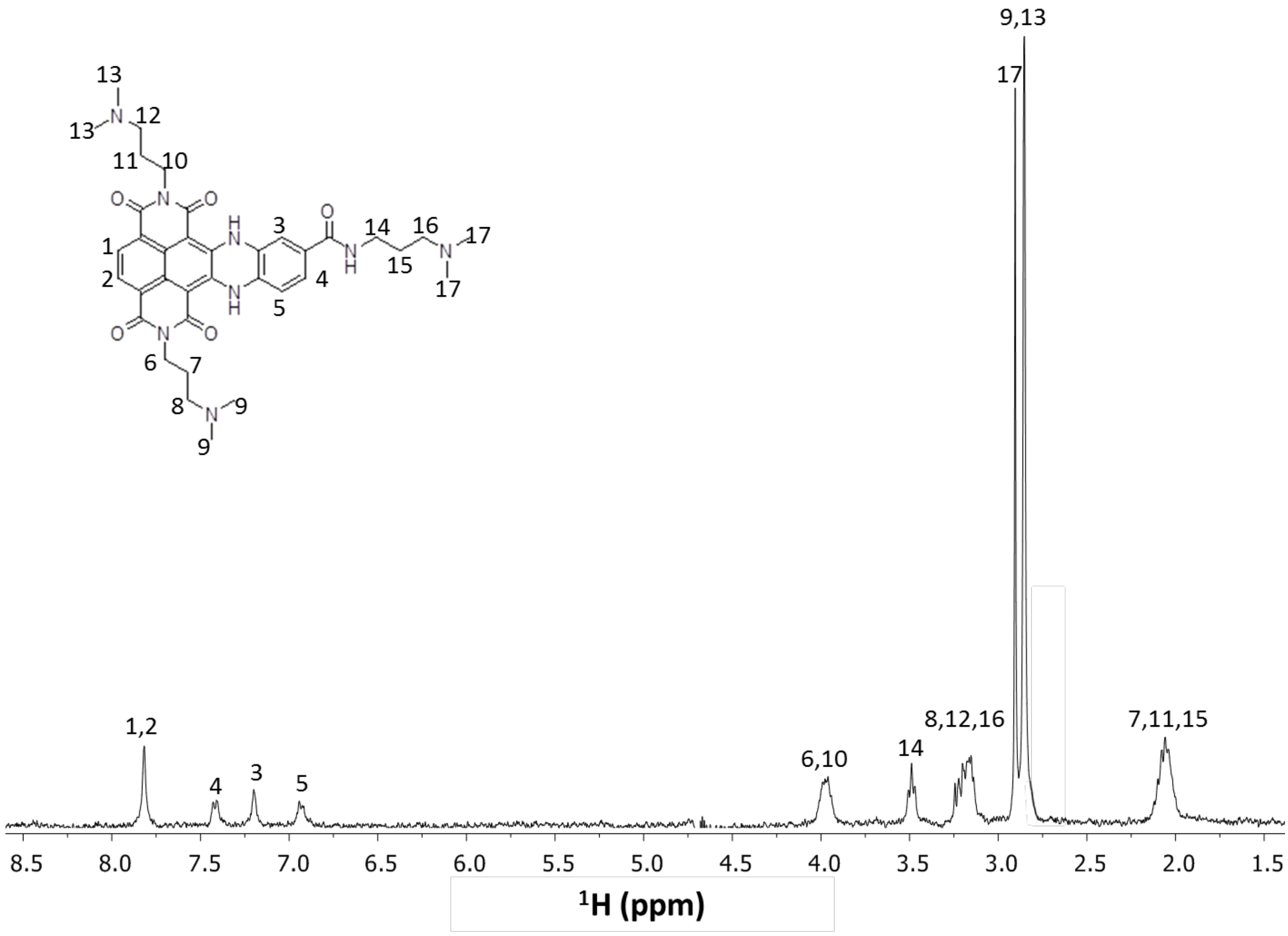

Figure S1. ¹H NMR spectrum of **c_{ex}-NDI** and related chemical structure and atom numbering used in the ¹H NMR assignments.

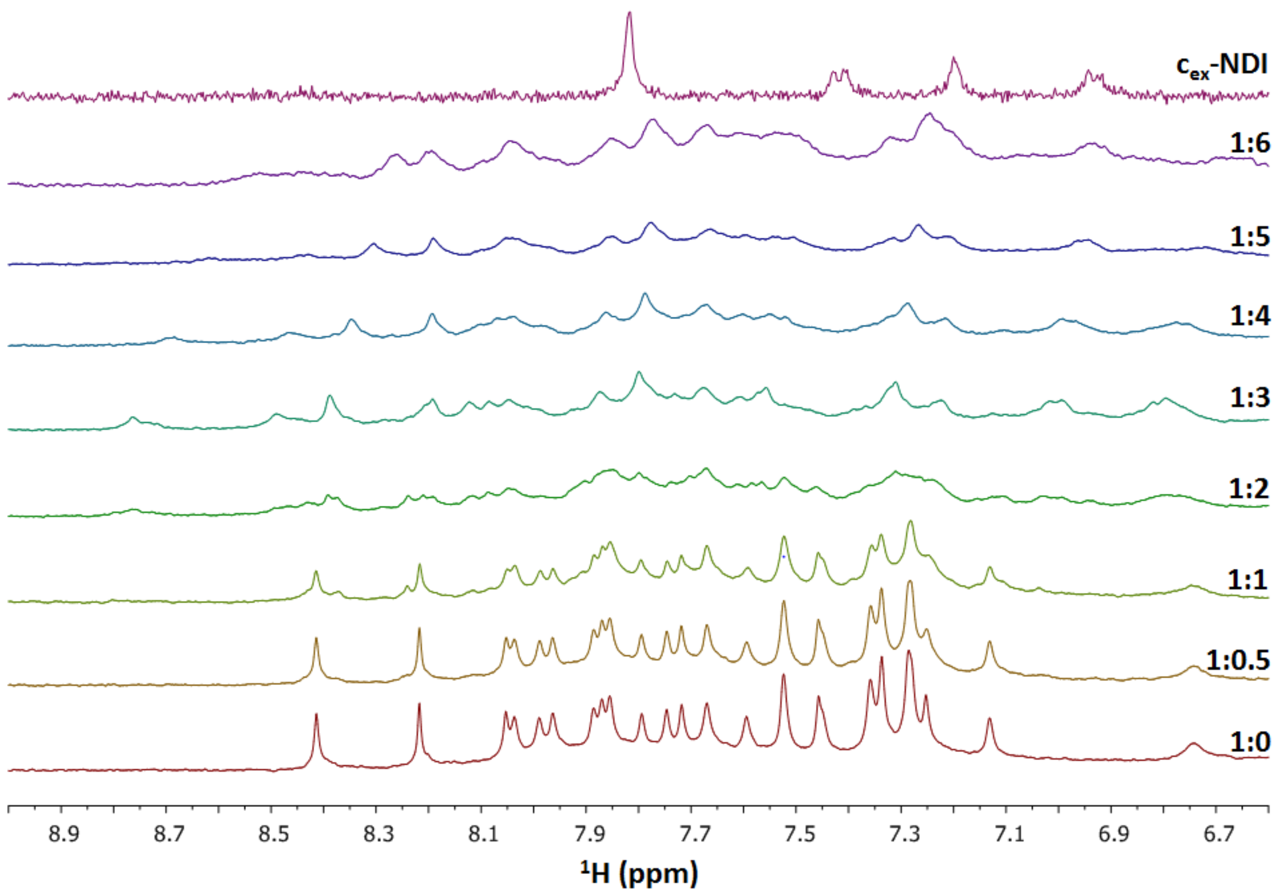

Figure S2. Aromatic proton regions of the ^{1}H NMR spectra of m-tel24 G-quadruplex upon titration with **c_{ex}-NDI** (from 0.5 to 6 equivalents). On top, NMR spectrum of free **c_{ex}-NDI** (0.2 mM) is reported. DNA/**c_{ex}-NDI** ratios are shown on the right of the corresponding spectrum.

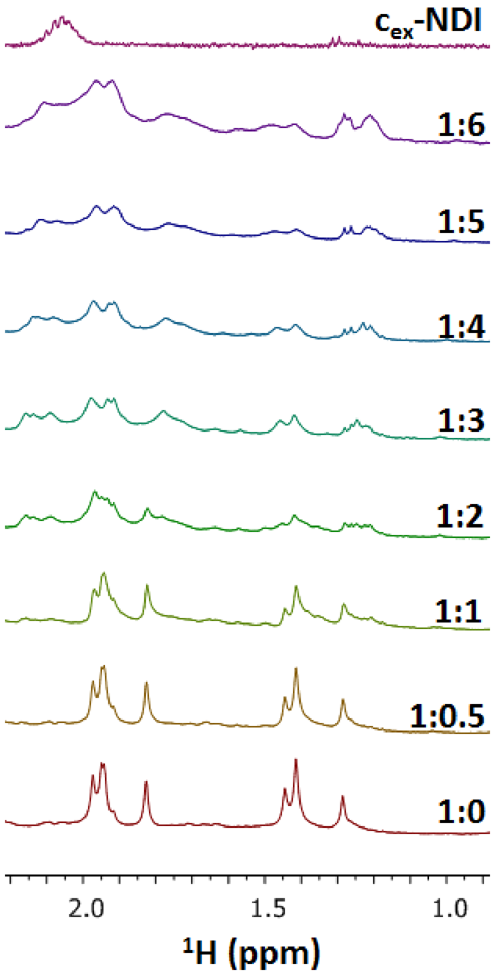

Figure S3. Methyl proton regions of the ^{1}H NMR spectra of m-tel24 G-quadruplex upon titration with **c_{ex}-NDI** (from 0.5 to 6 equivalents). On top, NMR spectrum of free **c_{ex}-NDI** (0.2 mM) is reported. DNA/**c_{ex}-NDI** ratios are shown on the right of the corresponding spectrum.

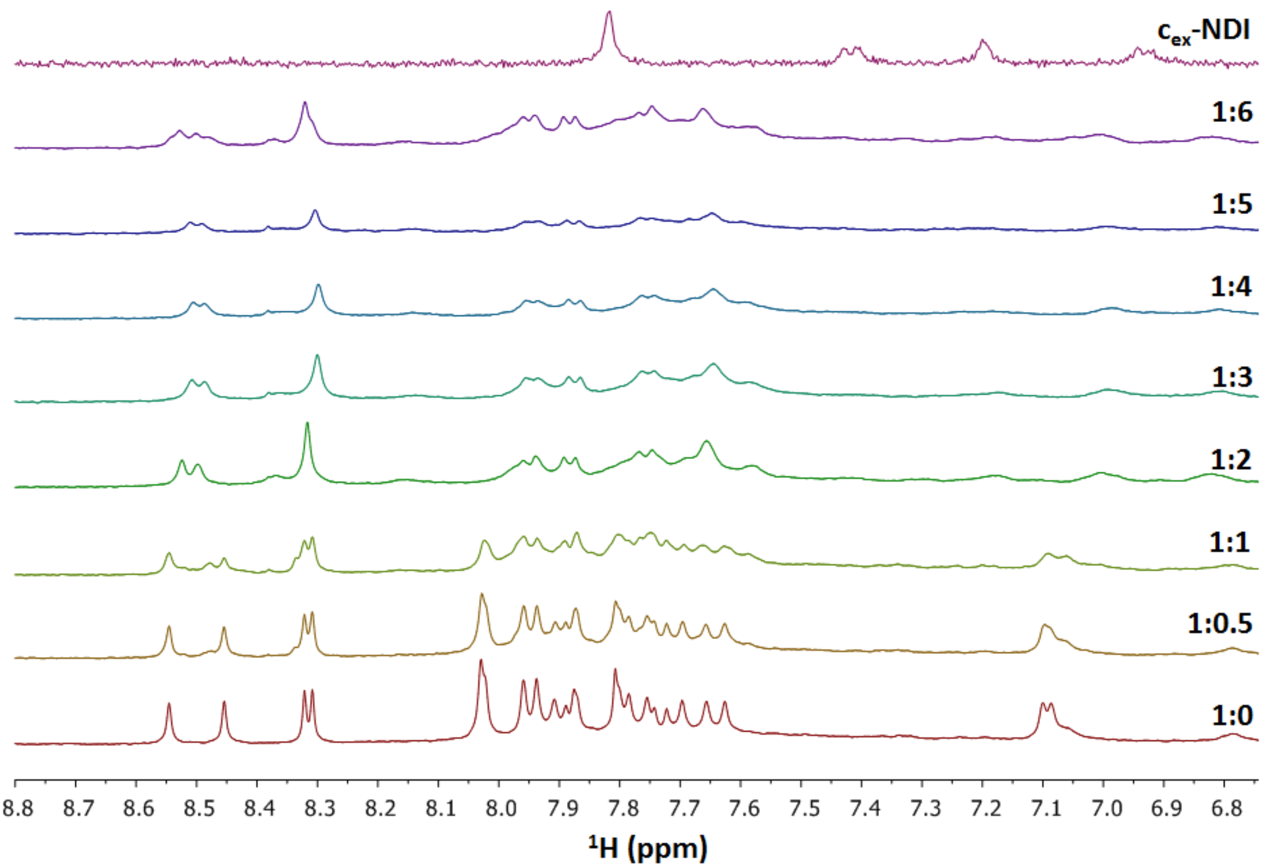

Figure S4. Aromatic proton regions of the ^{1}H NMR spectra of M2 G-quadruplex upon titration with **c_{ex}-NDI** (from 0.5 to 6 equivalents). On top, NMR spectrum of free **c_{ex}-NDI** (0.2 mM) is reported. DNA/**c_{ex}-NDI** ratios are shown on the right of the corresponding spectrum.

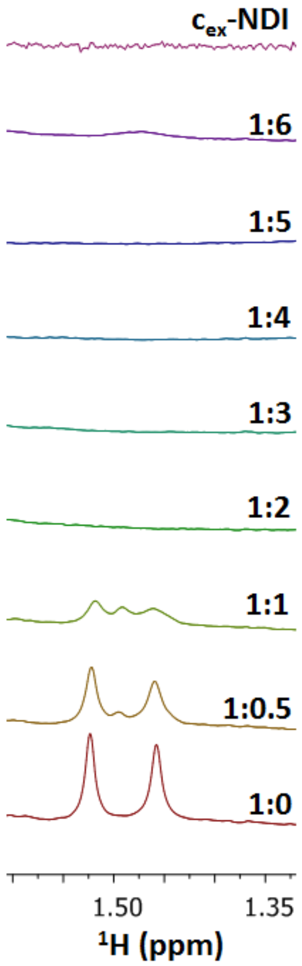

Figure S5. Methyl proton regions of the ^{1}H NMR spectra of M2 G-quadruplex upon titration with **c$_{ex}$-NDI** (from 0.5 to 6 equivalents). On top, NMR spectrum of free **c$_{ex}$-NDI** (0.2 mM) is reported. DNA/**c$_{ex}$-NDI** ratios are shown on the right of the corresponding spectrum.

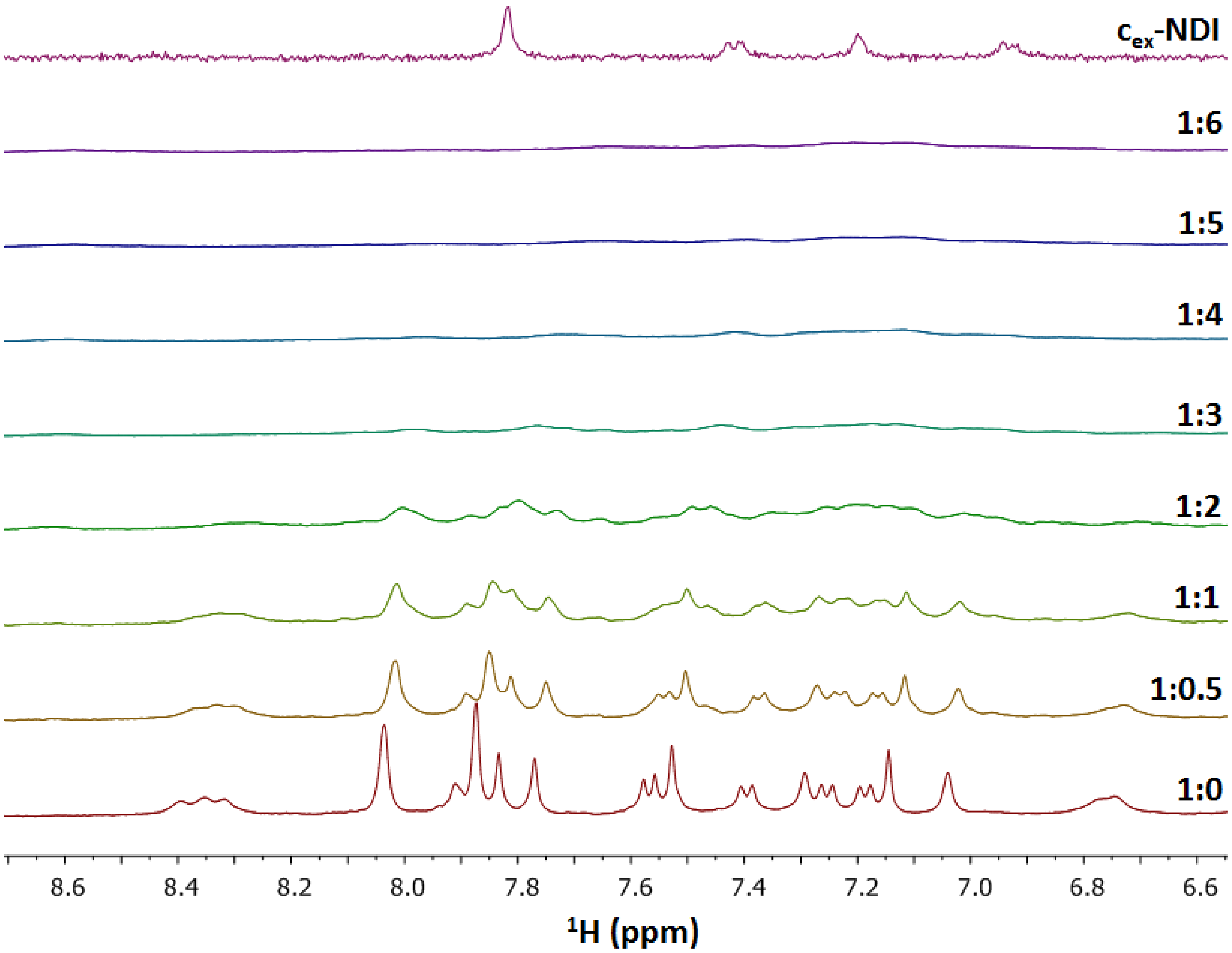

Figure S6. Aromatic proton regions of the ^{1}H NMR spectra of ds12 duplex upon titration with $\mathbf{c_{ex}}$**-NDI** (from 0.5 to 6 equivalents). On top, NMR spectrum of free $\mathbf{c_{ex}}$**-NDI** (0.2 mM) is reported. DNA/$\mathbf{c_{ex}}$**-NDI** ratios are shown on the right of the corresponding spectrum.

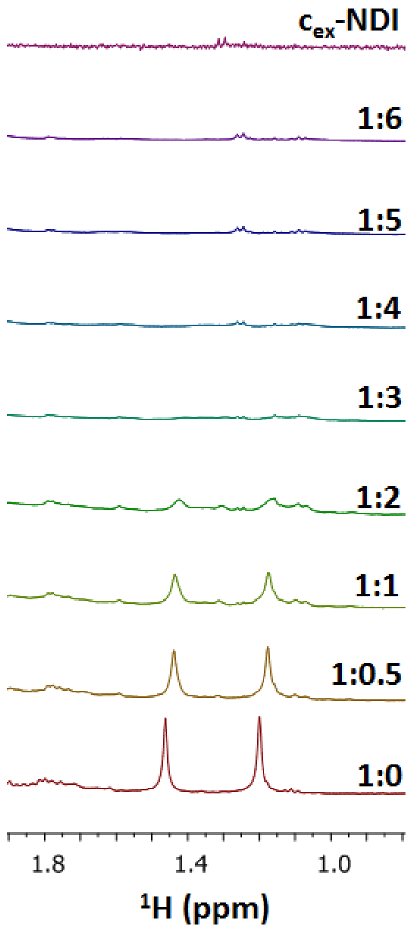

Figure S7. Methyl proton regions of the ^{1}H NMR spectra of ds12 duplex upon titration with $\mathbf{c_{ex}}$**-NDI** (from 0.5 to 6 equivalents). On top, NMR spectrum of free $\mathbf{c_{ex}}$**-NDI** (0.2 mM) is reported. DNA/$\mathbf{c_{ex}}$**-NDI** ratios are shown on the right of the corresponding spectrum.

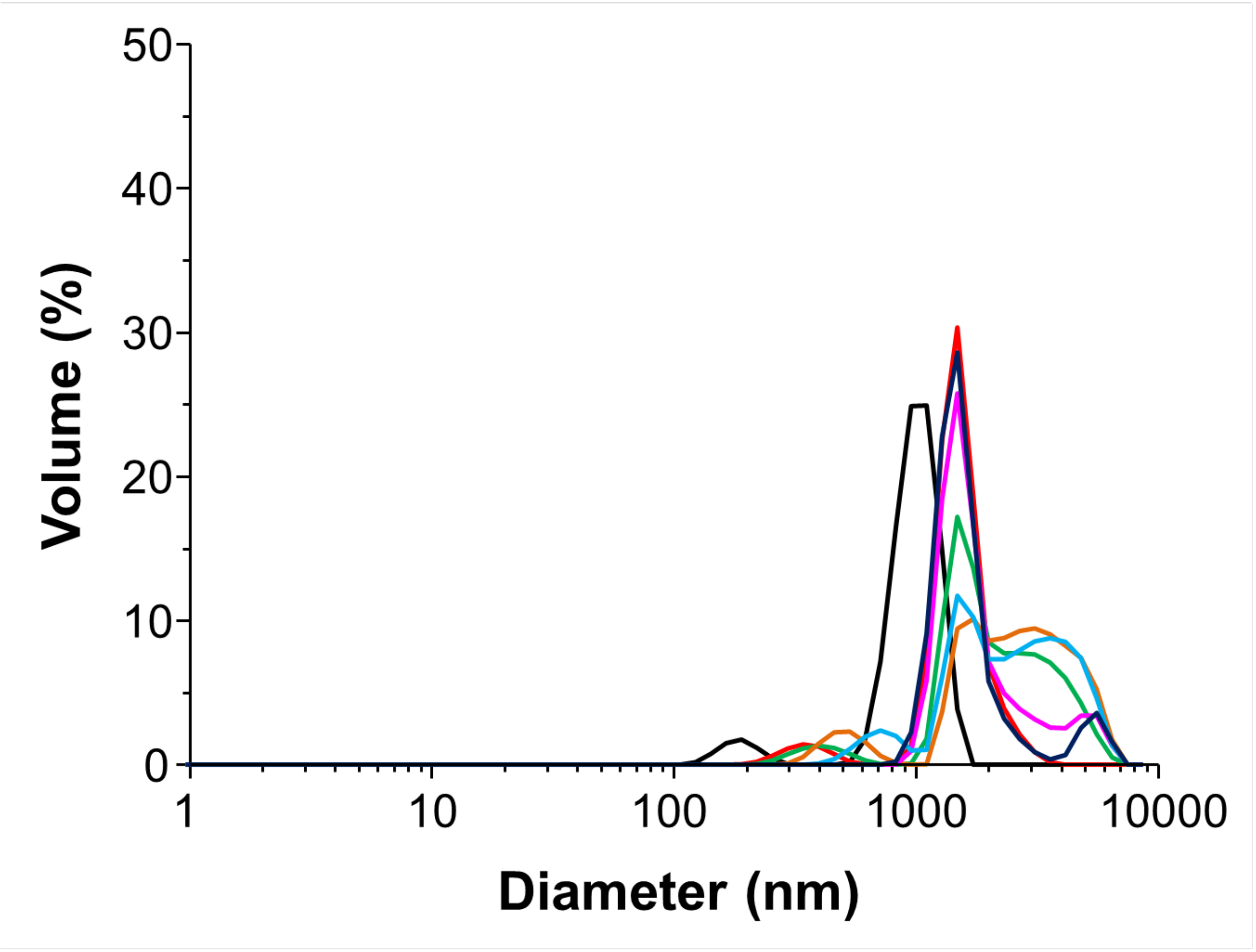

Figure S8. Volume-based particle size distribution for different amounts of $\mathbf{c_{ex}}$**-NDI** corresponding to 0.5 (black line), 1 (red line), 2 (blue line), 3 (fuchsia line), 4 (green line), 5 (cyan line) and 6 (orange line) ligand equivalents with respect to DNA in the DLS titration experiments.

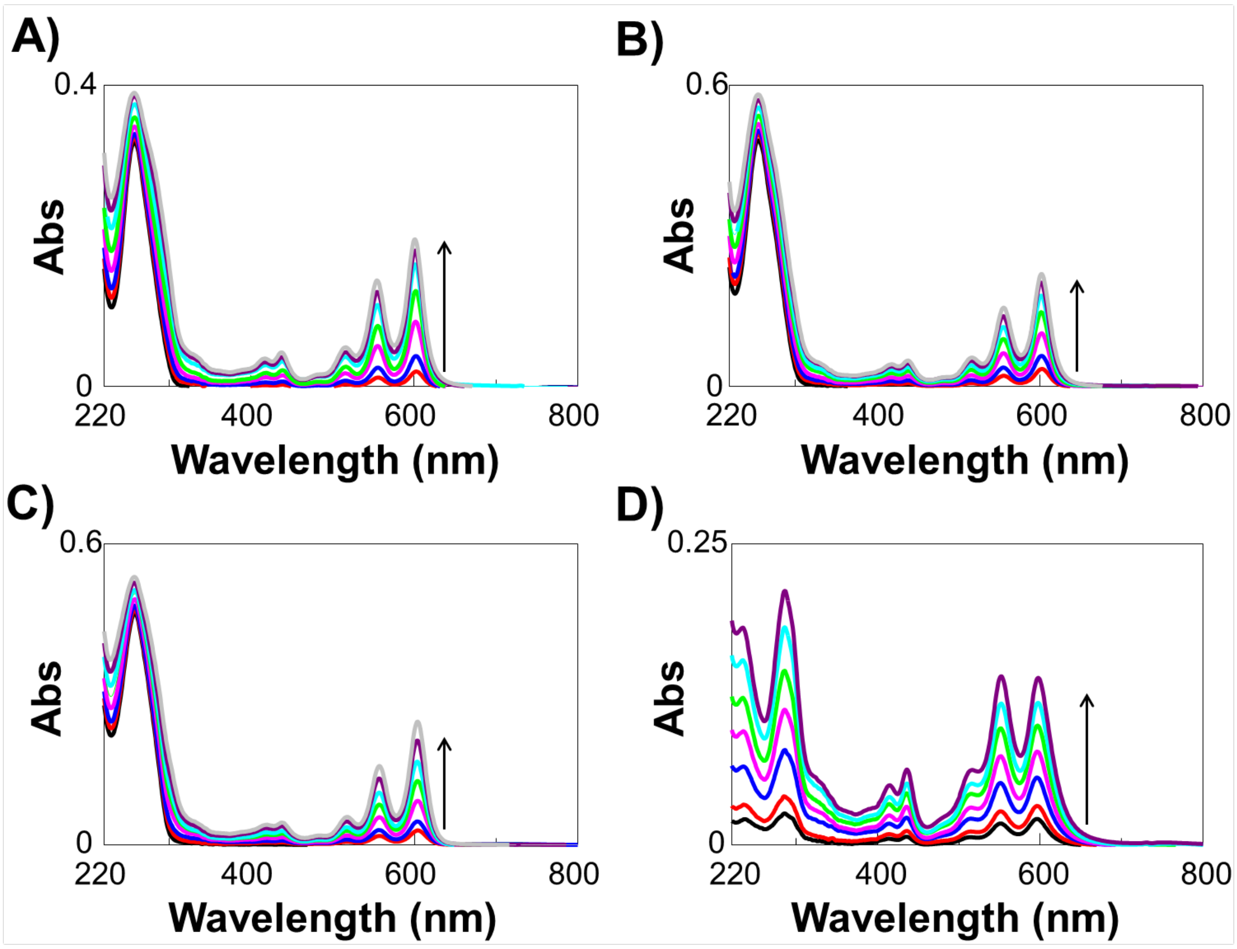

Figure S9. UV-vis spectra in 100 mM KCl and 20 mM potassium phosphate buffer (pH 7.0) of 2 μM solutions of A) m-tel24 G-quadruplex, B) M2 G-quadruplex and C) ds12 duplex in the absence or presence of increasing amounts (up to 6 molar equivalents) of $\mathbf{c_{ex}}$**-NDI** and D) free $\mathbf{c_{ex}}$**-NDI** at different concentration (from 1 up to 12 μM). The arrows indicate UV-vis band intensity variation on increasing $\mathbf{c_{ex}}$**-NDI** concentration.

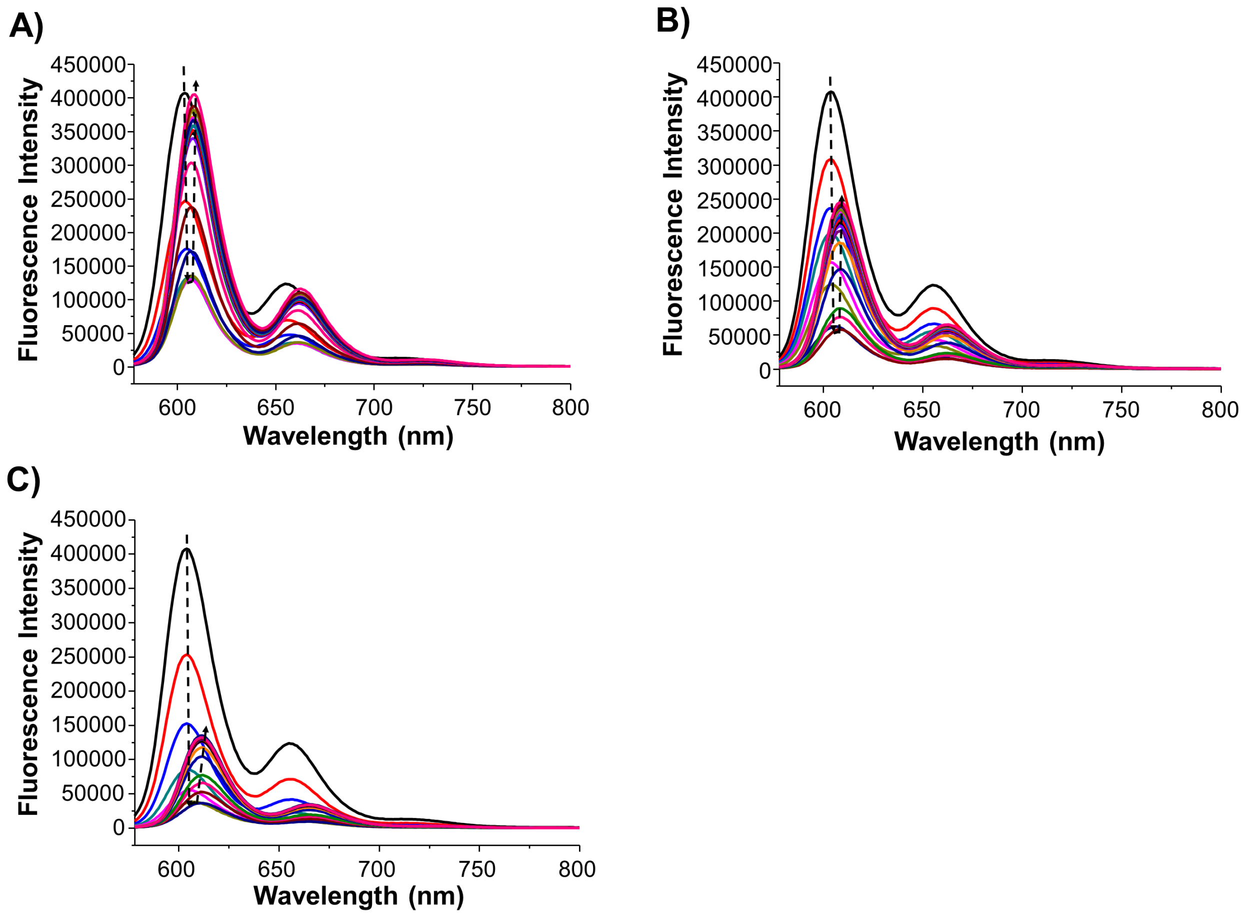

Figure S10. Fluorescence titration experiments of **c_{ex}-NDI** with A) m-tel24 G-quadruplex, B) M2 G-quadruplex and C) ds12 duplex. The arrows indicate fluorescence band intensity variation on increasing DNA concentration.

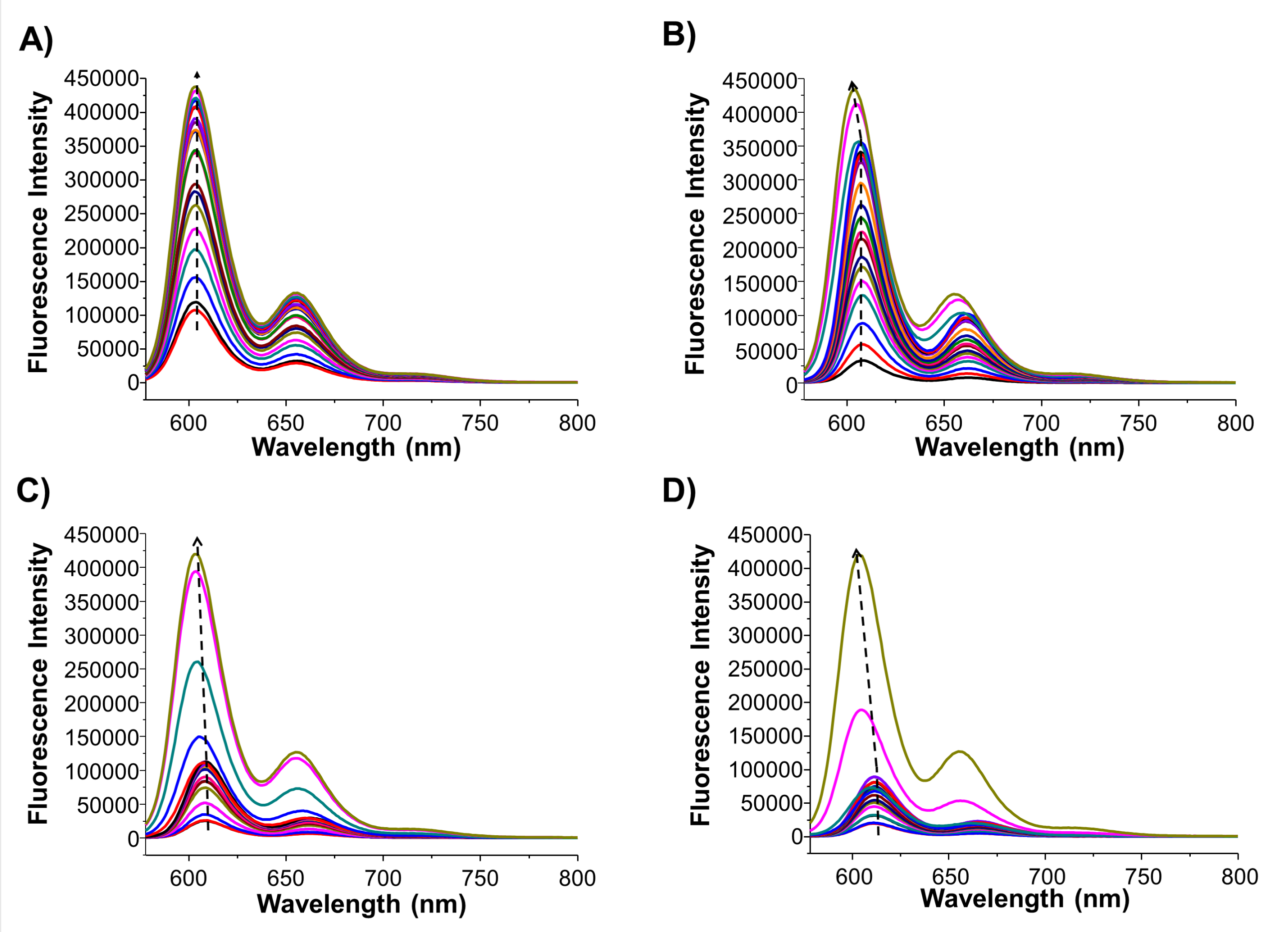

Figure S11. Fluorescence spectra of $\mathbf{c_{ex}}$**-NDI** in the A) absence and presence of B) m-tel24 G-quadruplex, C) M2 G-quadruplex and D) ds12 duplex recorded for Job plot analysis. The arrows indicate fluorescence band intensity variation on increasing $\mathbf{c_{ex}}$**-NDI** concentration.

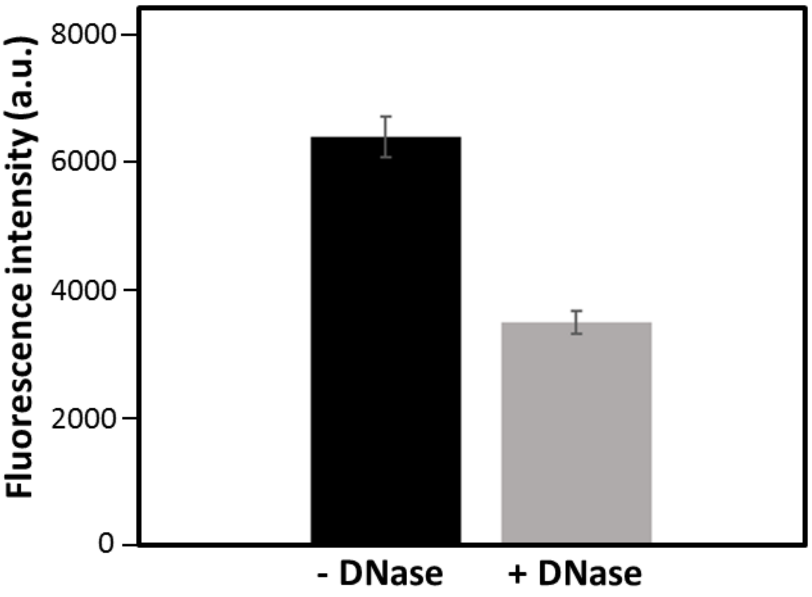

Figure S12. Fluorescence analysis of MCF7 cells incubated with 1.25 μM **c_{ex}-NDI** with or without a subsequent treatment with DNase. Quantification of the fluorescence signal intensity was performed with Zen Blue software on at least 6 different cell acquisitions.

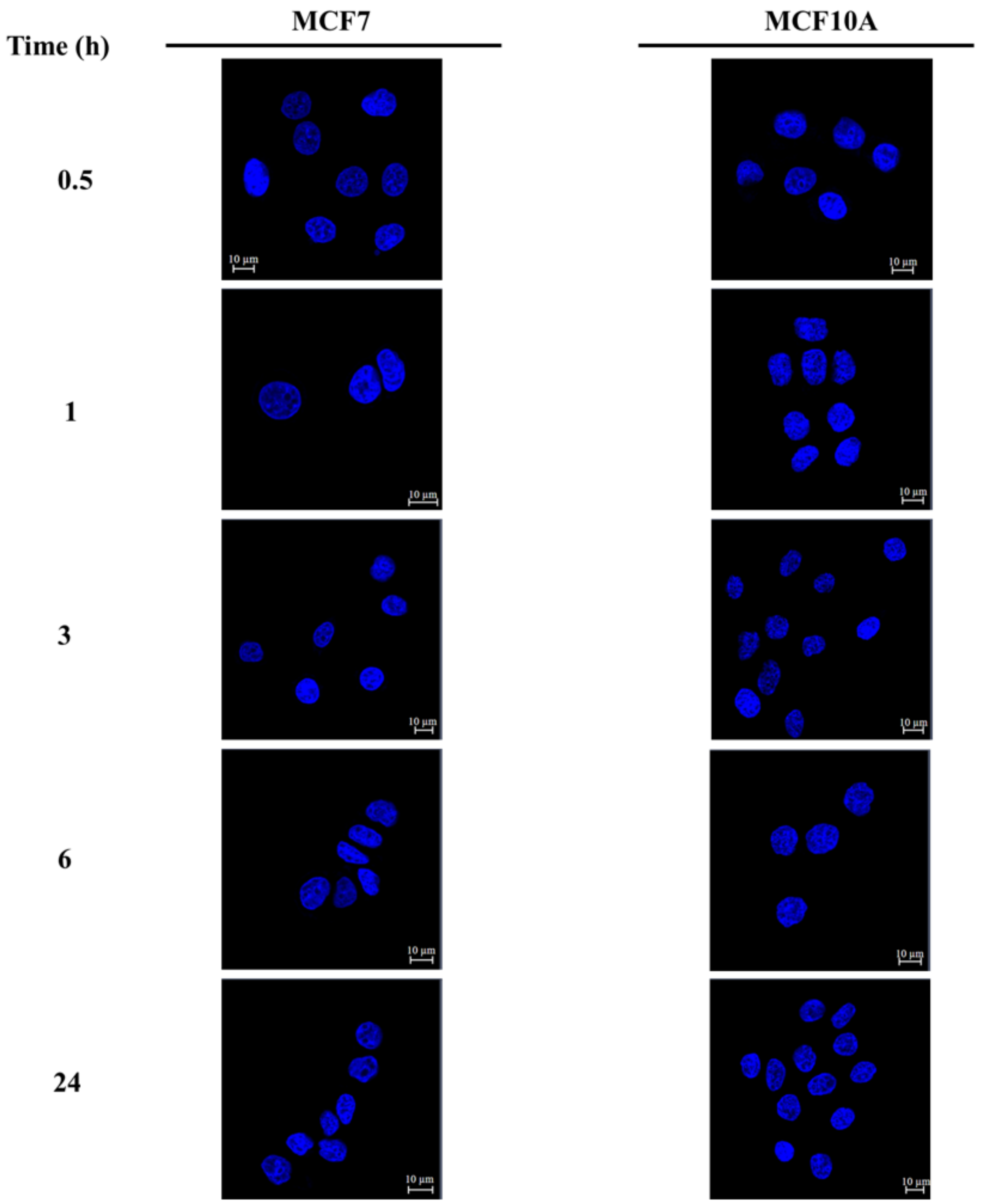

Figure S13. Confocal microscopy analysis of MCF7 and MCF10A cells. Cells were cultured on glass coverslips in 24-well plates, grown to semi-confluency and analysed after 0.5 - 1 - 3 - 6 - 24 h. Cell nuclei were stained by incubating the cells with 0.001 mg/mL Hoechst in PBS 1X for 20 min in the dark. After fixing the cells with 4% paraformaldehyde, they were analysed by CLSM by using a 63x oil immersion objective. Scale bars correspond to 10 µm.
